# Do health education initiatives assist socioeconomically disadvantaged populations? A systematic review and meta-analyses

**DOI:** 10.1186/s12889-023-15329-z

**Published:** 2023-03-08

**Authors:** E. L. Karran, A. R. Grant, H. Lee, S. J. Kamper, C. M. Williams, L. K. Wiles, R. Shala, C. V. Poddar, T. Astill, G. L. Moseley

**Affiliations:** 1 IIMPACT in Health, University of South Australia, GPO Box 2471, Adelaide, South Australia 5001 Australia; 2 Centre for Statistics in Medicine, Nuffield Department of Orthopaedics, Rheumatology and Musculoskeletal, Sciences, University of Oxford, Oxford, UK; 3 School of Medicine and Public Health, University of Newcastle, Newcastle, NSW Australia; 4 School of Health Sciences, University of Sydney, Sydney, NSW Australia; 5 Nepean Blue Mountains Local Health District, Penrith, NSW Australia; 6 University of Newcastle, Sydney, NSW Australia; 7 Population Health, Hunter New England Local Health District, New Lambton, NSW Australia; 8 Australian Institute of Health Innovation, Macquarie University, Sydney, NSW Australia; 9 South Australian Health and Medical Research Institute (SAHMRI), Adelaide, South Australia Australia; 10 Department of Physiotherapy, Faculty of Medicine, University of Prishtina, Prishtina, Kosovo; 11 Sir H. N. Reliance Foundation Hospital and Research Centre, Mumbai, India; 12 Western Sydney University, Sydney, NSW Australia

**Keywords:** Health education, Socio-economic disadvantage, Systematic review, Social determinants of health, Health promotion

## Abstract

**Background:**

Health education interventions are considered critical for the prevention and management of conditions of public health concern. Although the burden of these conditions is often greatest in socio-economically disadvantaged populations, the effectiveness of interventions that target these groups is unknown. We aimed to identify and synthesize evidence of the effectiveness of health-related educational interventions in adult disadvantaged populations.

**Methods:**

We pre-registered the study on Open Science Framework https://osf.io/ek5yg/. We searched Medline, Embase, Emcare, and the Cochrane Register from inception to 5/04/2022 to identify studies evaluating the effectiveness of health-related educational interventions delivered to adults in socio-economically disadvantaged populations. Our primary outcome was health related behaviour and our secondary outcome was a relevant biomarker. Two reviewers screened studies, extracted data and evaluated risk of bias. Our synthesis strategy involved random-effects meta-analyses and vote-counting.

**Results:**

We identified 8618 unique records, 96 met our criteria for inclusion – involving more than 57,000 participants from 22 countries. All studies had high or unclear risk of bias. For our primary outcome of behaviour, meta-analyses found a standardised mean effect of education on physical activity of 0.05 (95% confidence interval (CI) = -0.09–0.19), (5 studies, *n* = 1330) and on cancer screening of 0.29 (95% CI = 0.05–0.52), (5 studies, *n* = 2388). Considerable statistical heterogeneity was present. Sixty-seven of 81 studies with behavioural outcomes had point estimates favouring the intervention (83% (95% CI = 73%-90%), *p* < 0.001); 21 of 28 studies with biomarker outcomes showed benefit (75% (95%CI = 56%-88%), *p* = 0.002). When effectiveness was determined based on conclusions in the included studies, 47% of interventions were effective on behavioural outcomes, and 27% on biomarkers.

**Conclusions:**

Evidence does not demonstrate consistent, positive impacts of educational interventions on health behaviours or biomarkers in socio-economically disadvantaged populations. Continued investment in targeted approaches, coinciding with development of greater understanding of factors determining successful implementation and evaluation, are important to reduce inequalities in health.

**Supplementary Information:**

The online version contains supplementary material available at 10.1186/s12889-023-15329-z.

## Introduction

Health promotion and the prevention of ill-health via population and individual level interventions are key recommendations of the World Health Organization for the management of communicable and non-communicable diseases [1, 2]. Specific health education interventions are considered integral to system-wide public health strategies [3, 4]. Such educational interventions commonly aim to promote understanding about how behaviours impact health, and require individuals to have the capacity to acquire, understand and operationalize the content of health education in order to improve their health status [4, 5]. These capacities are influenced by the social and economic circumstances of individuals’ lives [6, 7].

Social and economic circumstances also importantly contribute to inequalities in health. This is depicted by the ‘social gradient’ in health, [8] whereby the lower a person’s socio-economic position, the poorer their health status. ‘Unhealthy’ behaviours associated with the development of chronic disease, such as smoking, poor diet, too little physical activity, and low engagement with preventative (e.g. screening) healthcare, are more prevalent among individuals who are socially or economically disadvantaged [9, 10]. Public health interventions to promote healthy behaviours may therefore be of most importance for these populations.

Socio-economically determined disparities in health outcomes can sometimes be further increased by behavioural health promotion initiatives, particularly those that are delivered across a large population. Benefit seems to be related to individuals’ access to social and economic resources and improvement is lowest in disadvantaged groups [10, 11]. For example, peoples abilities to respond to health promotion messages by changing health behaviours (such as improving diet and exercising regularly) vary widely – but changes are less likely to be adopted amonst low-income groups [10]. Similarly, technological interventions to improve health outcomes “work better for those who are already better off”(p. 1080), for reasons that stem from discrepancies in accessibility, adoption, and adherence [12]. Intensive, small-scale interventions targeted to high risk populations may be more likely to generate benefits, but economic and practical issues commonly limit broad implementation. Even the best-intentioned interventions frequently fail to reach, and to impact, those whose health needs are greatest.

Although specific educational interventions to improve health literacy and health-related behaviours are considered integral to public health interventions, little is known about the extent to which educational interventions that target disadvantaged populations are effective, nor about the intervention characteristics that are associated with success. Our principal objective was to identify and synthesize evidence of the effectiveness of health-related educational interventions in adult disadvantaged populations. Our primary outcome was health related behaviour, and our secondary outcome was a biomarker related to the health intervention. Our secondary objective was to summarise the characteristics of effective interventions.

## Methods

We registered our full protocol a priori on Open Science Framework (https://osf.io/ek5yg/). Our study is reported in accordance with the Preferred Reporting Items for Systematic Reviews and Meta-Analyses (PRISMA) statement, [13, 14] the Checklist of Items for Reporting Equity-Focused Systematic Reviews (PRISMA-E 20,212 Checklist), [15] and the Synthesis Without Meta-analysis (SWiM) [16] reporting guidelines. We deviated from the registered protocol by reconsidering our approach to addressing the secondary objective of this study and undertaking an additional vote-count analysis.

### Search strategy and selection criteria

We developed a comprehensive search strategy with the assistance of a health librarian and systematically searched five electronic databases (MEDLINE, EMBASE, EMCARE, and the Cochrane Central Register of Controlled Trials (CENTRAL)) since inception to 20^th^ May 2020 to identify eligible studies. We updated these searches on 5^th^ April 2022. Studies were limited to those involving human participants and available in English. Details of the search strategies are provided in Appendix 1.

We searched for studies that assessed the effectiveness of any health-related educational intervention delivered to socio-economically disadvantaged adults in any country. We defined *health* according to the World Health Organization definition, as: “a state of complete physical, mental and social well-being and not merely the absence of disease or infirmity” [17]. We defined *socio-economically disadvantaged adults* as belonging to a socio-economically disadvantaged population, classified as: “an area, neighbourhood or community with residents clearly defined as disadvantaged, relative to the wider national population” [18] (p. 372). Socio-economic disadvantage could be defined by factors including (but not limited to) income, educational level, living standards, and minority grouping. To be eligible for inclusion, at least 75% of participants in the included studies were required to meet this definition of belonging to a socio-economically disadvantaged population and be aged 18 years or over.

Published, peer-reviewed experimental studies investigating the effectiveness of an educational intervention on health-related outcomes were considered for inclusion. Eligible designs included (but were not limited to): randomised controlled trials, quasi-randomised and cluster-randomised trials. We excluded studies that were not published in English, pilot studies, reviews, commentaries, and case study reports, studies that did not describe the study population sufficiently to enable classification as ‘socio-economically disadvantaged’, and studies that did not report at least one outcome of interest.

### Interventions and outcomes

Studies included in this review must have evaluated the effectiveness of an educational intervention. Interventions were considered to be ‘educational’ if the authors described the intervention as having intent to ‘educate’ or ‘inform’. Studies evaluating an educational intervention as their main objective or as a component of a comprehensive intervention were eligible for inclusion. Individual, group, community or population-based health education interventions, delivered through any medium (e.g. face-to-face, telephone, text, online, mass media) were considered. Included studies needed to have compared the educational intervention to any type of intervention, placebo, or no-treatment control. The primary outcome was health-related behaviour, or actions that individuals take that affect their health [19]. All behavioural outcomes that were considered to be health related *and* related to the study intervention were regarded as relevant. The secondary outcome was any biomarker related to the health condition the intervention was targeting (e.g. body mass index (BMI) as a biomarker of weight loss; or Haemoglobin A1C as a biomarker of diabetes control).

### Screening and data extraction

Identified studies were retrieved and exported into Endnote citation management software (Clarivate Analytics, Philadelphia), and then imported into Covidence systematic review management system (Veritas Health Innovation Limited, Australia). Duplicates were removed. Pairs of reviewers independently screened all titles and abstracts for relevance according to the inclusion and exclusion criteria (AG, CP, TA, LW and RS). The full texts of potentially eligible studies were obtained, the article further screened for eligibility and reasons for exclusion recorded. Any discrepancies or disagreements between the two reviewers were discussed. If agreement was not met, a third reviewer (EK) was consulted to provide opinion and a majority decision was made.

Pairs of reviewers independently extracted the relevant data from each study using a standardised and pilot-tested spreadsheet. The results were compared, discrepancies discussed, and a third reviewer was consulted to resolve disagreements if required. The data extraction template included the fields: study design, health ‘condition’, population characteristics (including reason for classification as socio-economically disadvantaged), participant characteristics, sample size, details of study intervention(s) and comparator, assessment time points, outcomes, and results.

### Risk of bias assessment

Pairs of authors independently evaluated the risk of bias (ROB) for each study using the Cochrane Collaboration’s tool for assessing ROB in randomised trials [20]. Six domains were evaluated: selection bias, performance bias, detection bias, attrition bias, reporting bias, and ‘other’ bias. We used the guideline provided by the Cochrane Handbook to assess each item as high, low or unclear ROB. A third reviewer was consulted to resolve any disagreements between the independent evaluations if required. Overall ROB was also assigned according to the Cochrane Handbook. Low overall ROB was assigned for studies where all key domains were low risk; unclear overall ROB was assigned when key domains were either low or unclear; and high overall ROB was assigned when one or more of the key domains were assigned a high ROB.

### Data analysis

To address our primary aim – to identify and synthesize evidence of the effectiveness of health-related educational interventions in disadvantaged populations – we extracted effect sizes and precision estimates from the included studies where available. If an effect size was not reported we extracted the number of participants in each condition, the means and standard deviations of the observations (at the longest follow-up timepoint). We examined the clinical and methodological heterogeneity between the included studies to determine the appropriateness of combining the effect sizes to estimate an overall effect for our primary and secondary outcomes. To determine the appropriateness of data pooling we primarily considered homogeneity of outcomes, follow-up durations and comparison groups. In cases where studies were considered to be sufficiently (clinically and methodologically) homogenous for pooling, but data were missing, we contacted study authors to request the missing data. Authors were emailed, with a follow-up email sent two weeks later. In the case of no reply a further email was sent after another week, and if there was still no reply the data were not included. Random effects meta-analysis (DerSimonian and Laird model [21]) was conducted using Comprehensive Meta-Analysis software (version 3We evaluated the quality of the evidence of the included studies and rated the certainty of recommendations using the Grading of Recommendations Assessment, Development and Evaluation (GRADE) framework [22]. Publication bias was assessed by visual inspection of a funnel plot; Egger’s test was applied if there were 10 or more studies in the meta-analysis [23].

Since meta-analysis could only be performed on a proportion of the studies, we summarised the overall effectiveness of interventions for our primary and secondary outcomes using a vote-counting approach [20]. When studies specified a single primary outcome, we determined intervention benefit from that outcome. We classified ‘intervention benefit’ using a standardised binary metric assigned according to the observed direction of effect. This classification was based on the point estimate of effect, without consideration of statistical significance or the size of the effect. Studies with a point estimate of effect in favour of the intervention were counted as [1]; studies with a point estimate of effect in favour of the control were not counted. When studies had two or more outcomes, we applied a decision rule to identify a single outcome from which to classify intervention benefit (Appendix 2). We calculated the number of effects showing benefit as a proportion of the total number of studies and determined a confidence interval using the Agresti-Coull interval method recommended for large sample sizes [24]. We undertook a subsequent calculation in which we determined the proportion of effective interventions by classifying benefit (for the outcome of interest) according to the conclusions of the individual studies, rather than using the point estimate to indicate effect. This approach minimised the risk of an inflated vote-count result.

To address our secondary objective – to summarise the characteristics of effective interventions – we tabulated details of the intervention (setting, type, dose, description) in a format to facilitate reader interpretation. Classification of intervention dose [25] (as low, moderate, or high) considered intervention duration (in months), frequency (number of contacts), and amount (in hours) (see Appendix 3 for details). We aimed to provide a summary of the features of the effective interventions.

### Role of the funding source

The funder of this study played no role in the study design, data collection, data analysis, data interpretation, writing of the report or decision to submit the paper for publication.

## Results

**Fig. 1:**
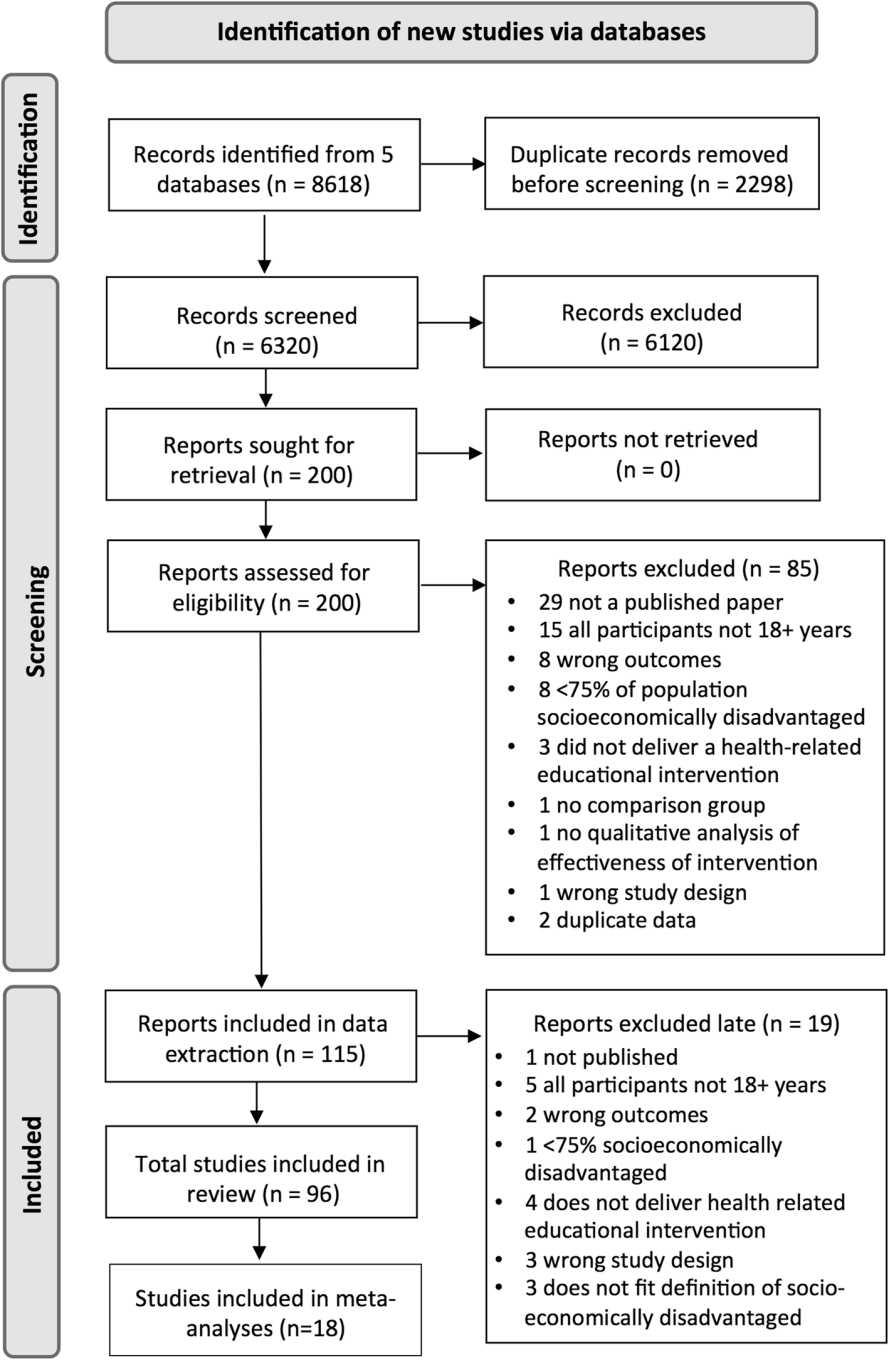
PRISMA flow chart

Our searches identified 8618 records; 200 full text articles were screened for eligibility; 96 studies were included (Fig. 1). Key characteristics of the included studies are provided in Tables 1 and 2. Eighty studies (83%) were undertaken in high-income countries; four studies (4%) were undertaken in upper-middle income countries; ten studies (10%) were undertaken in lower-middle income countries; and 3 studies (3%) were undertaken in low-income countries (see Tables 1 and 2). Seventy-seven (80%) of the included studies were randomised controlled trials (RCTs); 12 were cluster RCTs (13%); 7 were quasi-experimental studies (7%). The educational interventions addressed a wide range of health issues. The most common education topics were parenting skills, pregnancy and newborn health, (14 studies each) cancer screening, multi-factorial healthy lifestyle interventions (11 studies each), diet (9 studies), smoking cessation (8 studies) and sexual health (5 studies). The total number of adult participants exceeded 57,000, residing in 22 different countries.

**Table 1:** Characteristics of studies included in Meta-analyses (*n* = 16)

1^st^ Author (year), country	Study design N^o^ at baseline (N^o^ analysed)	Country (Income level)	Population and setting	Focus of educational intervention	Comparison group	Relevant Outcome(s)
Brooking (2012) [53]	RCT 84 (64)	New Zealand (HIC)	Maori at risk of type 2 diabetes	Weight loss and nutrition education	Control group with delayed educational content	Weight, BMI, BP (2), cholesterol (2), triglycerides, blood glucose, blood insulin
Byrd (2013) [32]	RCT 613 (613)	USA (HIC)	Women of Mexican origin from three diverse sites including a large urban centre and a rural farming community	Intervention to increase cervical cancer screening rates (3 intervention arms)	Usual care (offered intervention after completion)	Cervical cancer screening (1)
Gathirua-Mwangi (2016) [33]	RCT 244 (237)	USA (HIC)	African American women eligible for a free mammogram	Breast cancer screening educational intervention	Usual care (may have received postcard reminder to schedule mammogram)	Mammography adherence
Hovell (2008) [29]	RCT 151 (138)	USA (HIC)	Low-income, sedentary Latino women through a community-based clinic	Exercise and diet intervention involving education and aerobic dance	Control group—received information unrelated to exercise, diet or cardiovascular disease	Exercise (3), VO2 max, cholesterol (2)
Katz (2007) [34]	RCT 897 (775)	USA (HIC)	White, African American and native American women living in a rural count through a rural community	Lay health advisor education program focused on mammography and the benefits of early detection of breast cancer	Control group received delayed intervention	Cervical cancer screening
Keyserling (2008) [30]	RCT 236 (212)	USA (HIC)	Mid-life women attending a community health care centre serving low income, minority patients	Enhanced lifestyle intervention to improve physical activity and diet	Minimal intervention—single mail out of pamphlets on diet and physical activity	Physical activity (6), dietary risk assessment, carotenoid index, BP, cholesterol, weight
Khare (2012) [27]	RCT 833 (505)	USA (HIC)	Disadvantaged, low-income, uninsured or underinsured women (English speaking)	Cardiovascular disease risk factor screening and education intervention plus a 12-week lifestyle change intervention	Minimal intervention—screening and education without lifestyle change intervention	Dietary intake (3), physical activity (2), BP, cholesterol, blood glucose, BMI
Khare (2014) [28]	RCT 180 (67)	USA (HIC)	Disadvantaged, low-income, uninsured or underinsured women (Spanish speaking)	Cardiovascular disease risk factor screening and education intervention plus a 12-week lifestyle change intervention	Minimal intervention—screening and education without lifestyle change intervention	Physical activity (2), cholesterol (2), glucose, BMI
Kim (2014) [54]	RCT 440 (369)	USA (HIC)	Korean American seniors with high blood pressure through community-based churches and senior centres	Community based self-help behavioural intervention to address high blood pressure	Control group—received a brochure that listed available community resources	BP (3)
Kisioglu (2004) [55]	RCT 430 (400)	Turkey (UMIC)	Middle aged women of low socioeconomic status in the poor outskirts of the city	Blood pressure and obesity reduction intervention	Control group—no training	BMI, BP, physical activity (3)
Kreuter (2005) [35]	RCT 1227 (881)	USA (HIC)	Low-income African American women through urban public health centres	Intervention promoting use of mammography and increased fruit and vegetable intake	Usual care	Mammogram, dietary intake
Parra-Medina (2011) [31]	RCT 266 (151)	USA (HIC)	Low-income African American women at high risk for cardiovascular disease	Lifestyle intervention aimed to reduce dietary fat intake and increase moderate to vigorous physical activity	Standard care—behavioural counselling, assisted goal setting, educational materials	Physical activity (2), dietary intake
Staten (2004) [56]	RCT 326 (217)	USA (HIC)	Uninsured, primarily Hispanic women over 50 in the community	General health education intervention (2 arms)	Low intensity intervention – diet and physical activity counselling, referral to education classes	BMI, waist to hip ratio, BP, blood glucose, cholesterol, triglyceride levels, physical activity
Suhadi (2018) [57]	Cluster RCT 190 (182)	Indonesia (LMIC)	Low socioeconomic status, minority adults from 4 villages	Cardiovascular disease risk awareness and prevention intervention	Control group – monitoring of blood levels only	ASCVD risk, BP, BMI, blood sugar, cholesterol (2)
Valdez (2016) [36]	RCT 943 (727)	USA (HIC)	Low-income, Latina women	Cervical cancer education program	Standard care – received brochure on gynaecological cancer	Cervical cancer screening
Zoellner (2016) [26]	RCT 301 (296)	USA (HIC)	Low-income adults in 9 medically underserved rural regions	Intervention targeted decreasing sugar sweetened beverage consumption	Control—group based physical activity promotion intervention	Sugary drink intake, diet, physical activity (2), BMI, weight, cholesterol (3), triglycerides, glucose, BP (2)

**Table 2:** Characteristics of studies *not* included in Meta-analyses

1^st^ Author (year), country	Study design N^o^ at baseline (N^o^ analysed)	Country (Income level)	Population and setting	Focus of educational intervention	Comparison group	Relevant Outcome(s)
Abiyu (2020) [58]	Cluster RCT 612 mother-infant pairs (554)	Ethiopia (LIC)	Mothers with infants < 6 months old residing in rural communities in Ethiopia	Feeding behaviour change intervention to improve infants’ feeding practices, health and growth	Usual care (routine health and nutrition services)	WHO dietary adequacy indicators (3), dietary intake (8)
Acharya (2015) [59]	Cluster RCT 12,368 (11,885)	India (LMIC)	Community-dwelling pregnant women in Uttar Pradesh districts (high socioeconomic needs and low institutional delivery)	Pregnancy and Newborn Health – High intensity intervention	Low intensity intervention	Healthy delivery (5); breast feeding(4)
Almabadi (2021) [60]	RCT 579 (295)	Australia (HIC)	Adults on a waiting list at an Oral Health Care Clinic in a low socio-economic community	Promoting improved oral health care via education about oral hygiene procedures, smoking and alcohol cessation, healthy diet	Routine oral health care	Smoking, alcohol, diet, BMI, blood makers (6), plaque index
Alegria (2014) [61]	RCT 724 (647)	USA (HIC)	Low-income Latino and/or other minority patients of community mental health clinics; English and Spanish speaking	Teaching activation, self-management, engagement & retention in mental healthcare	Minimal intervention (received brochure)	Patient activation, self-management, service use, retention
Alias (2021) [62]	Quasi-experimental 390 (358)	Spain (HIC)	Community dwelling older adults (≥ 60 years) living in urban disadvantaged areas who perceived their health as fair or poor	Aimed at promoting social support and participation, self-management and health literacy	Delayed intervention	Social participation; use of anxiolytics/antidepressants; use of health resources
Alvarenga (2020) [63]	RCT 56 (44)	Brazil (UMIC)	Mother-infant dyads recruited from 2 health centres in 2 low-income communities	Infant development	Control intervention (monthly mailouts showing main developmental milestones)	Mother behaviours related to maternal sensitivity (6)
Andrews (2016) [64]	RCT 409 (373)	USA (HIC)	Female smokers residing in government subsidized neighbourhoods in South Carolina	Smoking cessation intervention	Delayed intervention group	Smoking cessation (2)
Annan (2017) [65]	RCT 479 (479)	Thailand (LMIC)	Burmese migrant parents or primary caregivers and their children residing in rural, peri-urban, or urban communities in Thailand	Parenting and family skills training program	Waiting list control condition	Child behaviour (3)
Avila (1994) [37]	RCT 44 (39)	USA (HIC)	Obese, low-income Latina from a community medical clinic	Weight reduction program including exercise, nutrition education, behavioural modification strategies, and a buddy system	Control intervention—weekly cancer screening education sessions	Exercise frequency, BMI, cholesterol, blood glucose, BP, VO2 max
Bagner (2016) [66]	RCT 60 families (46)	USA (HIC)	Racial minority mothers and their 12–15-month-old infants living below the poverty line	Parenting intervention involving an Infant Behaviour Program	Standard paediatric care	Parent child interaction (2)
Baranowski (1990) [67]	RCT 94 families (94)	USA (HIC)	Black American families with children in 5^th^, 6^th^ and 7^th^ grade in community-based public or private school systems	Centre-based program to improve diet and increase aerobic activity	No intervention control group (no contact during the program)	Exercise (2), resting pulse rate, BP
Barry (2022) [68]	RCT 574 (364)	USA (HIC)	English-speaking mother-infant dyads living in poverty in one of two major US cities	Positive parenting and healthy child development	Usual care	Child behaviour (4), continuous performance task
Befort (2016) [69]	RCT 172 (168)	USA (HIC)	Postmenopausal female breast cancer survivors residing in rural areas through rural community cancer clinics	Diet and physical activity intervention (Phase 2—weight maintenance intervention)	Minimal intervention – mailout and phone calls covering the same educational content	Weight (4)
Berman (1995) [70]	Quasi-experimental 446 (118)	USA (HIC)	Adult smokers who were parents of students or adult students from low to middle income, multi-ethnic, inner-city public-schools	Smoking cessation program	Control group—received health education material without smoking cessation information	Smoking cessation (4)
Bray (2013) [71]	Quasi-experimental 727 (727)	USA (HIC)	Rural, low income, diabetic African Americans in rural, fee for service primary care practices	Diabetes self-management program involving education, self-management coaching and medication adjustment	Usual care—standard assessment and treatment, educational handouts offered	Haemoglobin, BP, lipid levels
Brooks (2018) [72]	Cluster RCT 331 (250)	USA (HIC)	Smokers interested in quitting smoking from Boston public housing developments	Smoking cessation intervention	Standard care—smoking cessation materials and one visit from a Tobacco Treatment Advocate	Service use; smoking cessation (2)
Brown (2013) [73]	RCT 252 (109)	USA (HIC)	Impoverished Mexican Americans with type 2 diabetes in the community	Culturally tailored diabetes self-management education intervention	Waiting list control	Leptin, A1C, BMI
Cahill (2018) [74]	RCT 267 (240)	USA (HIC)	Socioeconomically disadvantaged pregnant African American women, overweight/obese before pregnancy	Homebased lifestyle weight management intervention to reduce gestational weight gain	Control group – parenting skills program	Weight (2), body composition, plasma glucose (2), insulin (2), lipids,
Calderon-Mora (2020) [44]	Cluster RCT 300 (257)	USA (HIC)	Underserved Hispanic women—uninsured or underinsured/low income/low educational attainment	Group cervical cancer screening education program	Individual counselling with identical education content	Cervical cancer screening (1)
Childs (1997) [75]	RCT 1000 (455)	England (HIC)	Children recorded on a child health register from households in inner city areas of high socioeconomic deprivation	Dietary health education program. Families received specific health education information at key child ages	Standard care	Haemoglobin; diet (2); breast feeding (3); intro-duction of pasteurised milk
Cibulka (2011) [76]	RCT 170 (146)	USA (HIC)	Low-income pregnant women in an inner-city hospital based prenatal clinic	Oral care education program and provision of dental supplies	Control group – education without dental supplies	Brushing & flossing, sugary drink intake, dental check up
Curry (2003) [77]	RCT 303 (ITT: 303)	USA (HIC)	Ethnically diverse, low-income female smokers whose children received care in a paediatric clinic	Smoking cessation intervention	Usual care with no education related to smoking cessation	Smoking cessation (4)
Damush (2003) [78]	RCT 211 (139)	USA (HIC)	Low income, inner city primary care patients with acute low back pain in an inner-city neighbourhood health centre	Acute low back pain self-management program	Usual care—referrals and analgesics as indicated, and back exercise sheets	Physical activity (4)
Dawson-McClure (2014) [79]	RCT 1050 (1050)	USA (HIC)	Low-income families with a non-Latino Black child in a pre-k program in disadvantaged urban neighbourhoods in New York City	ParentCorps Intervention aimed to increase parent involvement in early learning and behaviour management	ParentCorps intervention not provided in control schools	Parenting practices (4)
Dela Cruz (2012) [80]	RCT 5,807 (5,807)	USA (HIC)	Low-income families with young children enrolled in Medicaid or Basic Health Plus in Yakima County, Washington State	Dental health care education	No postcard mailings	Service use
Doorenbos (2011) [43]	RCT 5605 (5363)	USA (HIC)	Urban, low-income American Indians and Alaska native patients through mail to patients of an urban American Indian clinic	Mail-out intervention to increase cancer screening	Mailed calendar without cancer screening messages	Smoking cessation, cancer screening (3)
El-Mohandes (2003) [81]	RCT 286 (167)	USA (HIC)	Lo- income minority mothers from a community-based hospital	Parenting skills education program	Standard social services care	Service use (2)
El-Mohandes (2010) [82]	RCT 691 (691)	USA (HIC)	Pregnant African American women from 6 clinics in Washington, DC	Intervention aimed at reducing environmental tobacco smoke exposure	Routine prenatal care	Environmental tobacco smoke exposure (2)
Emmons (2001) [83]	RCT 291 (279)	USA (HIC)	Low-income smokers or recent quitters through community-based health centres	Intervention for smoking parents of young children aimed at reducing household passive smoke exposure	Self-help smoking cessation resources	Household nicotine levels
Falbe (2015) [84]	RCT 55 parent–child dyads (41)	USA (HIC)	Overweight or obese Latino parent and child dyads using federally funded care	Obesity intervention (Active and Healthy Families Intervention)	Usual care wait list control condition	BMI (2), BP, lipids, blood glucose, insulin (2), haemoglobin A1C
Fernandez-Jimenez (2020) [85]	Cluster RCT 635 parent–child dyads (446)	USA (HIC)	Low-income and minority parents or caregivers and their children from 15 Head Start preschools in Harlem, New York	Health promotion intervention (2 arms) to improve cardiovascular risk factor profiles (Peer-to-Peer Program)	Control group received education unrelated to cardiovascular health	Composite health score, FBS
Fiks (2017) [86]	RCT 87 (71)	USA (HIC)	Low-income, Medicaid insured new mothers of infants at high risk of obesity	Intervention to address parenting, maternal wellbeing, feeding and infant sleep	No education—text message appointment reminders only	Infant feeding, sleep, activity; maternal well being
Fitzgibbon (1996) [87]	RCT 38 families (36)	USA (HIC)	Low-income inner city Hispanic American families living in the community in Chicago	Dietary intervention to reduce cancer risk	Control received health related pamphlets	Parent support, diet intake (2), BP
Fitzgibbon (2004) [41]	RCT 256 (195)	USA (HIC)	Latino women from the Erie Family Health Centre	Combined dietary and breast health intervention	Control group received health information unrelated to breast health	Breast self-examination (2)
Fox (1999) [88]	RCT 646 (566)	USA (HIC)	Residents in 9 rural counties with a minimum of 15% of their population below the poverty line and 10% minority population	‘In-home’ mental health screening and educational intervention	Control group—received list of local resources for health/mental health care	Rates of help seeking behaviour
Gielen (1997) [89]	RCT 467 (391)	USA (HIC)	Low income, minority pregnant women smokers from an urban prenatal clinic	Smoking cessation and relapse prevention program (Smoke-Free Moms Project)	Usual care – routine clinic and inpatient smoking cessation education	Smoking cessation
Hayashi (2010) [40]	RCT 1093 (869)	USA (HIC)	Low-income, uninsured/underinsured Hispanic women at risk for cardiovascular disease	Lifestyle intervention to improve health behaviours and reduce cardiovascular disease risk factors	Usual in-clinic care only with no lifestyle intervention	Eating habits (3); physical activity (3); BP; BMI; CHD risk; cholesterol; smoking
Hesselink (2012) [90]	Quasi-experimental 239 (183)	Nether-lands (HIC)	1^st^ and 2^nd^ generation Turkish women living in the Netherlands through parent–child centres providing integrated maternity and infant care	Antenatal education program	Usual care	Smoking during pregnancy, parenting behaviours (2)
Hillemeier (2008) [39]	RCT 692 (362)	USA (HIC)	Low socioeconomic status women, pregnant or able to become pregnant in low income urban, rural and semirural locations	Health education intervention to improve health behaviours and health status of pre-conceptional and inter-conceptional women	Control group	Physical activity, reading food labels, multivitamin use, BMI, weight, BP, blood glucose, cholesterol
Hoodbhoy (2021)	Cluster RCT 32,595	Pakistan (LMIC)	Pregnant women and their families residing in a rural low-resource setting	Maternal and perinatal health program aimed at reducing all-cause maternal and perinatal morbidity and mortality	Routine antenatal	Birth preparedness (composite score & individual items (6))
Hooper (2017) [91]	RCT 342 (282)	USA (HIC)	Low-income African American smokers through a university	Smoking cessation intervention	Standard CBT intervention—not culturally based	Smoking cessation
Hunt (1976) [92]	RCT 344 (200)	USA (HIC)	Low-income pregnant women of Mexican descent from Los Angeles County prenatal clinics	Nutrition education intervention	Control group given vitamin and mineral capsules but no education	Dietary nutrients from blood samples (12)
Jacobson (1999) [93]	RCT 433 (318)	USA (HIC)	Inner city minority patients 65 + years, presenting for routine primary care at an inner-city public hospital	One-page, low literacy patient education tool encouraging patients to ask their doctor about pneumococcal vaccination	Control group—one-page handout about nutrition	Vaccination rates, vaccination discussions with clinician
Janicke (2008) [94]	RCT 93 (71)	USA (HIC)	Families with overweight children in underserved rural settings through Cooperative Extension Service offices	Diet and exercise intervention (two arms)	Waiting list control	Child’s BMI
Jensen (2021) [95]	Cluster RCT 149814981498149814981498149814981498149814981498(1354)	Rwanda (LIC)	Families belonging to the most extreme level of poverty with one or more children aged 6–36 months	Early childhood development and non-violence	Usual care including social protection public works program and government support services	Violence and safety, harsh discipline
Kalichman (2000) [42]	RCT 105 (53)	USA (HIC)	Inner city, low-income African American women who were patients of a community-based health clinic	Breast self-examination skills building workshop	Control group—sexually transmitted diseases prevention workshop	Breast self-examination skills and rate
Kasari (2014) [96]	RCT 147 (95)	USA (HIC)	Families low income or with mothers with low educational attainment, or a primary carer who is unemployed, in low resource communities	Caregiver-mediated intervention for pre-schoolers with Autism	Two active interventions compared: individual and group	Parent–child interaction (4)
Kelly (1994) [97]	RCT 197 (93)	USA (HIC)	Low-income, minority women in neighbour-hoods with high rates of sexually transmitted diseases, drug abuse & teenage pregnancy	HIV and AIDS risk reduction group education	Control group received sessions on health topics unrelated to AIDS	Safe sex practices (9)
Kim (2021) [98]	RCT 63 (56)	South Korea (HIC)	Low-income women (40–60 years) residing in J Provence, South Korea	Healthy lifestyle intervention addressing nutrition, exercise, stress, psychological distress and dementia prevention	Minimal intervention (booklet with diet and exercise advice)	Health promoting behaviour, BMI, % body fat, waist-hip ratio
King (2013) [38]	RCT 40 (39)	USA (HIC)	Low-income, inactive older adults through community centres serving primarily Latino population in San Jose, California	Physical activity intervention	Control group—received information about non-physical activity topics	Physical activity
Kreuter (2010) [45]	RCT 489 (429)	USA (HIC)	Low-income African American women through low-income community neighbourhoods	Breast cancer screening intervention	Content equivalent video using a more explanatory and didactic approach	Mammogram
Krieger (2005) [99]	RCT 274 (214)	USA (HIC)	Low-income, ethnically diverse urban households in their homes	High intensity intervention to decrease exposure to indoor asthma triggers	Low intensity intervention group	Asthma trigger reduction behaviour
Kulathinal (2019) [100]	Quasi-experimental 405 (380)	India (LMIC)	Married men and women from primary health centres in rural Western India	Sexual and reproductive health intervention	Control areas received no mobile helpline	Contraceptive use (2)
Lutenbacher (2018) [101]	RCT 188 (178)	USA (HIC)	Low-income pregnant Hispanic women in isolated community in a large metropolitan area	Home visiting program using peer mentors to improve maternal and child health outcomes	Minimal education intervention group received printed educational materials only	Breast feeding (3), prenatal care visits, reading stories, infant sleeping (2)
Maldonado (2020) [102]	Quasi-experimental 379 (326)	Kenya (LMIC)	Pregnant women attending their first antenatal care visits at a public health facility in a rural sub-county in Kenya	Education addressed antenatal care, family planning, intimate partner violence and microfinance literacy	Standard care (no structured education)	Facility-based delivery, healthy parenting practices (4), financial planning
Manandhar (2004) [103]	Cluster RCT 24 clusters (24)	Nepal (LMIC)	Poor married women of reproductive age in a community based rural district	Childbirth and care behaviours intervention	Health service strengthening activities only	Antenatal care (10)
Martin (2011) [104]	RCT 434 (338)	USA (HIC)	Low income, rural adults receiving medication at no charge from a public health department or a federally funded rural health centre	Adherence to hypertensive medications intervention	Control group – received cancer information	Medication adherence
McClure (2020) [105]	RCT 718 (526)	USA (HIC)	Socioeconomically disadvantaged English- speaking adults who smoked > 5 cigarettes/day and were ready to quit smoking	A novel oral health and smoking cessation program	Standard smoking cessation program	Smoking cessation, oral health behaviours (4)
McConnell (2016) [106]	RCT 104 (59)	Kenya (LMIC)	New mothers from a peri-urban community	Postnatal care intervention (2 arms)	Standard care group	Vaccination, family planning, breast feeding (2), index of health practices
McGilloway (2014) [107]	RCT 149 (137)	Ireland (HIC)	Families in an urban disadvantaged area defined by their demographic profile, social class composition, and labour market situation	Parenting intervention aimed at fostering positive parent child relationships	Waiting list control	Child conduct (2), service use (2), social competence
Miller (2013) [108]	RCT 210 (82)	USA (HIC)	Inner city, low income, minority women who had an abnormal pap smear	Colposcopy appointment adherence intervention (2 arms)	Enhanced standard care—included appointment reminders	Colposcopy (2)
Murthy (2019) [109]	Quasi-experimental 2016 (1417)	India (LMIC)	Low-income pregnant women in urban slums (selected based on being in slums that are high proportion low income)	Healthy infant intervention	Control group	Child immunization, healthy infant nutrition (7)
Pandey (2007) [110]	Cluster RCT 1045 households (1025)	India (LMIC)	Low socioeconomic status, resource poor, rural village clusters in Uttar Pradesh through the community	Pre-natal and infant health care utilisation	Control village clusters receiving no intervention	Prenatal care (3), tetanus injection, infant received vaccination
Phillips (2014) [111]	RCT 53 (53)	Australia (HIC)	Australian Aboriginal children with tympanic membrane perforation through remote communities	Child ear health intervention	Usual care – received information sheet, treat-ment guidelines, advice to attend weekly clinic	Service use
Pitchik (2021) [112]	Cluster RCT 621 (568)	Bangla-desh (LMIC)	Pregnant women or primary caregivers of a child < 15 months residing in rural villages	Child development intervention including caregiver behaviours, nutrition, caregiver mental health and lead exposure prevention	No intervention	Stimulation in the home
Polomoff (2022) [113]	RCT 188 (180)	USA (HIC)	Cambodian Americans aged 35–75 years at high risk of developing diabetes and meeting the criteria for likely depression	A bilingual, trauma-informed, cardio-metabolic education intervention to decrease diabetes risk	Control intervention (needs assessment and support)	Medication forgetting
Reijneveld (2003) [114]	RCT 126 (92)	Nether-lands (HIC)	Turkish immigrants aged 40 + years old recruited via welfare services	Health education and physical exercise program	Control group received the ‘Ageing in the Netherlands’ program	Physical activity
Reisine (2012) [115]	RCT 120 (93)	USA (HIC)	Low-income pregnant women attending a community health centre	Dental caries prevention and nutrition education	Control group – received dental caries prevention education only	Mutans levels, Service use, teeth brushing
Ridgeway (2022) [116]	RCT 1377 (943)	USA (HIC)	Women 40–74 years presenting for a screening mammogram at a health clinic serving a primarily Latina/Latino population	Education to explain the meaning and implications of mammographic breast density	Usual care (mailed mammogram results only)	Provider conversations relating to breast density
Robinson (2002) [117]	RCT 218 (122)	USA (HIC)	Low-income African American women	HIV and sexually transmitted diseases prevention intervention combined with comprehensive sexuality education	Control group—received an HIV pamphlet and a gift card to a local beauty school	Sexual communication (3)
Ryser (2004) [118]	RCT 54 (54)	USA (HIC)	Low-income pregnant women	Breast feeding education program	Control group—no exposure to Best Start program	Breast feeding
Saleh (2018) [119]	RCT Data from 2359 patient records	Lebanon (UMIC)	Individuals with noncommunicable diseases in rural areas and refugee camps	Hypertension and diabetes self-management education	Control group—no intervention	BP (2), diabetes markers (3)
SantaMaria (2021) [120]	RCT 519 (397)	USA (HIC)	Parents of caregivers of youth 11–14 years of age living in medically underserved communities	Sexual health intervention including adolescent vaccinations and HPV	Control intervention – received nutrition and exercise information	Vaccination initiation and completion
Segal-Isaacson (2006) [121]	RCT 466 (230)	USA (HIC)	Women with HIV/AIDS	High intensity coping skills, stress management and nutrition education intervention	Low intensity intervention—education with no individualization	CD4 and CD8 cell count, viral load, lipids
Seguin-Fowler (2020) [122]	Cluster RCT 182 (182)	USA (HIC)	Women aged ≥ 40 years who were overweight or obese and sedentary; lived rurally in medically underserved towns	Healthy lifestyle intervention to reduce risk for cardiovascular disease	Delayed intervention	smoking cessation, diet, physical activity, weight, blood pressure, cholesterol, blood glucose
Simmons (2022) [123]	RCT 1467 (1417)	USA (HIC)	Hispanic and Latino smoking adults	Smoking cessation program	Usual care (mailed Spanish language quit smoking booklet)	Smoking cessation
Smith (2021) [124]	RCT 240 (240)	USA (HIC)	Racially and ethnically diverse low-income families with an overweight child attending paediatric primary care	Parenting skill development, connection with community-based services, telephone/face-to-face coaching	Usual care (information about services)	Child physical activity, diet, BMI, mealtime/media/sleep routines
Steptoe (2003) [125]	RCT 271 (218)	USA (HIC)	Low-income, minority patients in a deprived ethnically diverse inner-city area	Individualised behavioural dietary counselling intervention targeted increasing intake of fruit and vegetables	Low intensity intervention—brief nutrition counselling	Dietary intake (2), nutrition blood levels (5), body weight, BMI, BP, cholesterol
Wiggins (2005) [126]	RCT 731 (601)	England (HIC)	Low-income, inner city, culturally diverse minority women with infants in two disadvantaged inner-city boroughs of London	New mothers support interventions (2 arms)	Low intensity intervention—routine health visiting services	Smoking, infant feeding
Xu (2019) [127]	RCT 278 (278)	Indonesia (LMIC)	Resource poor villagers diagnosed with schizophrenia in 9 rural townships	Schizophrenia support intervention (Lay health supporters, E-platform, Aware and iNtegration (LEAN))	Usual care – included a public health program for people with psychosis	Medication adherence

### Risk of bias

**Fig. 2:**
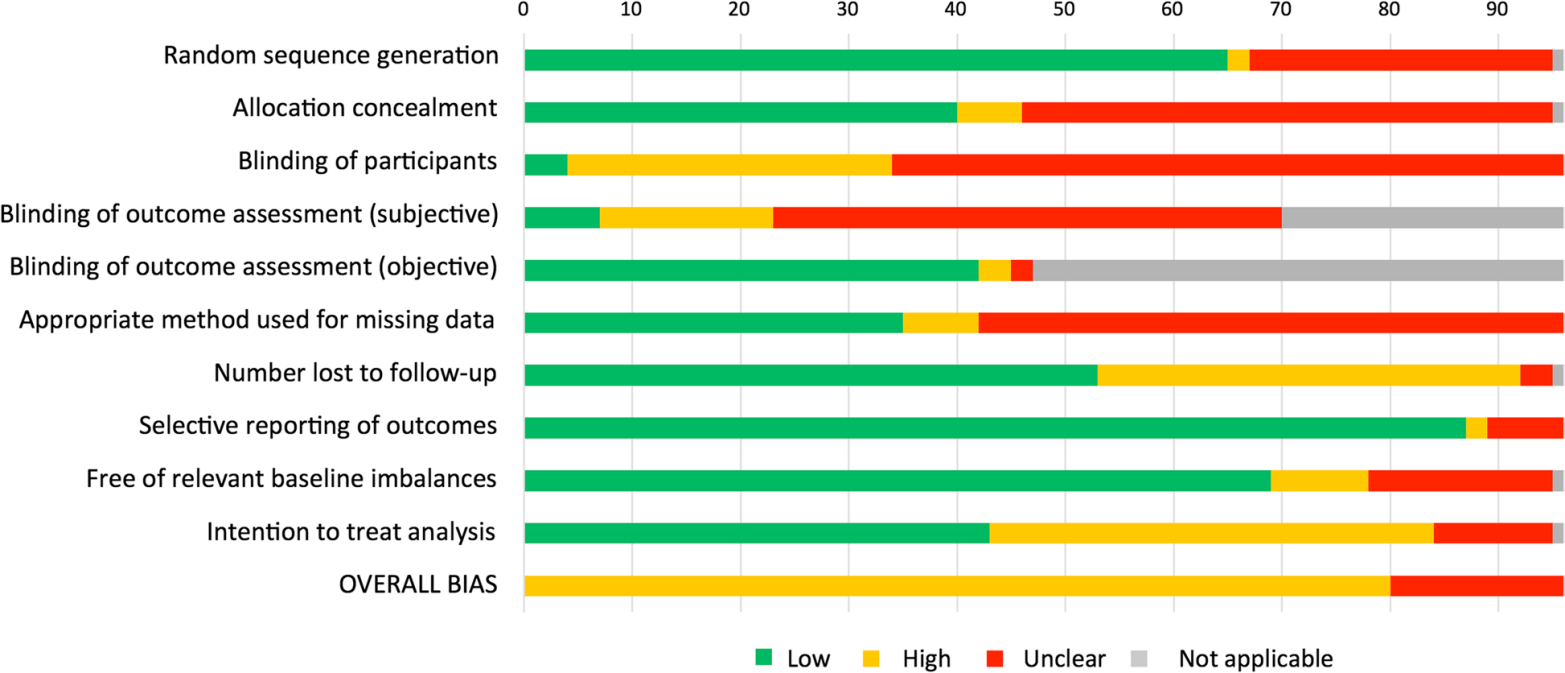
Risk of bias summary

All included studies had either high or unclear overall ROB. The ‘other’ ROB domain of ‘intention to treat analysis’ was most frequently assessed as high. High ROB ratings were also common for ‘number lost to follow up’ and participant blinding (Fig. 2; see Appendix 4 for full details). Visual inspection and interpretation of the funnel plots for each main meta-analysis (to evaluate publication bias) identified no major asymmetries in the distribution of effects for any of the outcomes (Appendix 5), suggesting a low risk of publication bias. Egger’s tests were not conducted because there were < 10 studies in each analysis [23].

### Certainty in evidence

Our evaluation of certainty in the evidence for each main meta-analysis was conducted using GRADE. Our results are summarised in relation to each meta-analysis (below); detailed results are provided in Appendix 6.

### Data synthesis

High clinical and methodological heterogeneity amongst the included studies precluded overall meta-analysis of effect sizes for the primary and secondary outcomes of this review. Instead, we considered outcomes that were evaluated in three or more of the included studies for meta-analysis. Pre-planned subgroup analyses (specified in the protocol) were explored for intervention complexity, the level of intervention and intervention dose.These were undertaken if there were two or more studies in a subgroup. Results of the main meta-analyses of behaviour outcomes are detailed below; results of subgroup analyses and the meta-analyses of biomarker outcomes are detailed in Appendices 7–9.

#### Meta-analyses: Behavioural outcomes

Fifteen studies had physical activity or exercise outcomes; nine had dietary outcomes; eight had smoking cessation outcomes; seven had cancer screening outcomes; and five had vaccination and breast-feeding outcomes. Meta-analysis was not conducted for studies involving dietary, smoking cessation, vaccination, and breast-feeding outcomes because of varied study designs, outcome measures, follow-up durations and comparison groups.

### Moderate intensity physical activity

**Fig. 3:**
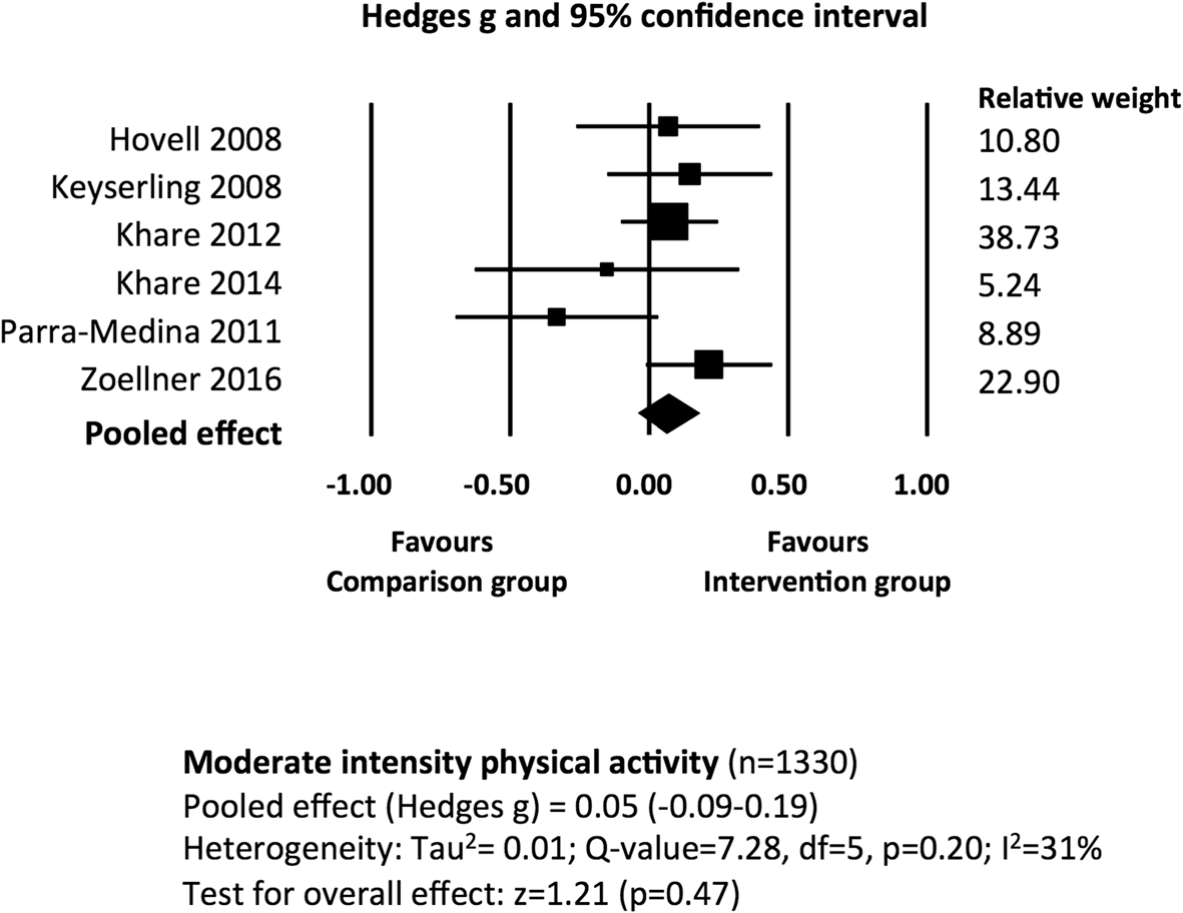
The effectiveness of educational interventions at improving moderate intensity physical activity outcomes in socio-economically disadvantaged populations: random effects meta-analysis

We evaluated the 15 studies with physical activity or exercise outcomes for clinical heterogeneity. Six of these studies (total *n* = 1330) used ‘moderate intensity physical activity’ as a primary or secondary outcome; the intervention group was compared with a minimal intervention, standard care or control group; and effectiveness was evaluated at ‘long term’ follow up [26–31]. We downgraded certainty in the evidence by one level due to high risk of bias. There is moderate certainty that the pooled effect of educational interventions, when compared to standard care, minimal intervention or control, is 0.05 (95% CI = -0.09–0.19; Tau^2^ = 0.01%) (Fig. 3). There was moderate heterogeneity (I^2^ = 31%), which we explored by removing one study that used a differing outcome measure (i.e. the percentage of participants who improved their physical activity in contrast to post-intervention physical activity measures) from the analysis (2011) [31]. This reduced I^2^ to 0.0% and the pooled effect increased to 0.11 (95% CI = -0.01–0.22). Subgroup analysis of studies with complex or ‘non-complex’ interventions were possible; the results are reported in Appendix 7.

### Cancer screening

**Fig. 4:**
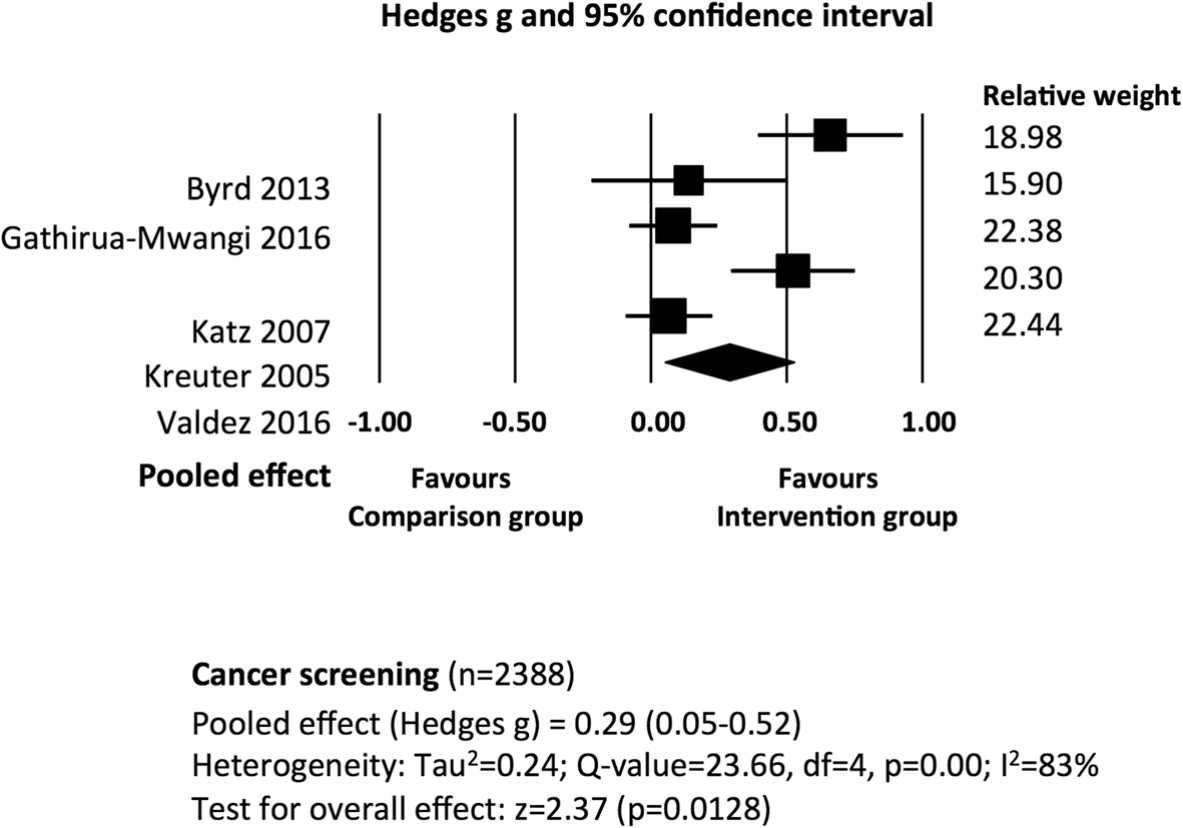
The effectiveness of educational interventions at improving cancer screening outcomes in socio-economically disadvantaged populations: random effects meta-analysis

We evaluated for clinical heterogeneity the ten studies that had cancer screening outcomes. Five of these studies (*n* = 2388) used rates of cancer screening as their primary or secondary outcome; the intervention group was compared with a minimal intervention, standard care or control group; and effectiveness was evaluated at ‘long term’ follow up [32–36]. We downgraded certainty in the evidence by four levels due to risk of bias, inconsistency (two levels), and imprecision in trial results. There is very low certainty that the pooled effect of educational interventions, when compared to standard care or minimal intervention is 0.29 (95% CI = 0.05–0.52; Tau^2^ = 0.24) (Fig. 4). The I^2^ value of 83% indicates a considerable degree of heterogeneity across trial results. We explored this heterogeneity by removing individual studies from the analysis, which had only a minor impact. Removal of one study [32] reduced statistical heterogeneity to a small degree (I^2^ = 75%). Subgroup analysis of studies with moderate or low-dose interventions were possible; the results are reported in Appendix 8.

### Overall synthesis: Vote-counting

We performed separate vote-counting syntheses for the behavioural outcomes and biomarker outcomes. Vote counting based on direction of effect found that 67 of the 81 studies with behavioural outcomes had point estimates that favoured the intervention (83% (95% CI 73%-90%), *p* < 0.001); ten studies favoured the control, and four studies demonstrated equal effects for intervention and control conditions. Twenty-one of 28 studies with biomarker outcomes had point estimates that favoured the intervention (75% (95% CI 56%-88%), *p* = 0.002); four studies favoured the control. Calculation of votes based on ‘effectiveness’ being determined by individual studies found 47% of interventions were effective on behavioural outcomes, and 27% were effective on biomarker outcomes. The votes assigned to each study by both vote-count methods are presented alongside the available data and/or effect estimates in Table 3.

**Table 3:** Intervention characteristics and effectiveness

**1**^**st**^** Author (year)**	**Health condition**	**Setting**	**Intervention summary**^**d**^	**Intervention description**	**Outcomes** **Bold text = behavioural** Plain text = biomarker	**Available data** *(Italics* = *calculated from reported data)*	**Stand. Metric** b	**VC**_**C**_ ^c^
Alegria (2014) [61^a^	Mental health	Outpatient health clinics	Education only Moderate dose Short term f/u	DECIDE Intervention: 3 x (30–45 min) didactic presentations sessions with opportunities for participation, role-play & reflection. Delivered in person or (rarely) by telephone over 3 months	**Self-management**	β(SE) = 2.42 (SE 0.90), d = 0.22	**1**	**1**
Fox (1999) [88]	Mental health	Home- based	Education ± PS Low dose Short term f/u	Single education session delivered with or without a significant other present. Involved a 1-h interview of 90 min duration (including a video) and a follow up phone call. Provided resource list of local mental health services	**Rates of help seeking behaviour**	(n = 566) Yates corrected χ^2^ (1) = 0.977, *p* = 0.32; favours intervention	**1**	**NS**
Xu (2019) [127]	Mental health	Home- based	Education + rewards High dose Medium f/u	LEAN intervention: 2 text messages (at 9am and 7 pm) per day for 6 months, send by an e-platform to the patient and to the lay health supporter, Lay health worker reviewed the patient on a 1:1 basis to ensure medication adherence and monitoring	**Medication adherence**	Mean difference 0.12 (95% CI 0.03 to 0.22)	**1**	**1**
Annan (2017) [65]^a^	Parenting skills	Home- based	Education only High dose Short term f/u	Instruction of parenting skills & social skills (children), practice of positive family interactions. 14 × weekly (in-person) education sessions, 2-h duration each, culturally adapted for non-literate participants. Integrated social learning theory	**Child attention problems**	Intervention 0.50 (SD 0.18); Control 0.52 (SD 0.26), ES = -0.23	**1**	**1**
Bagner (2016) [66]	Parenting skills	Home- based	Education only Moderate dose Long term f/u	Parenting intervention program with education and problem-solving skills training. Up to 7 × weekly one-on-one sessions delivered to caregiver (until caregiver meets mastery), 1 to 1.5 h duration	**Observed parent 'don't' skills**	Intervention (*n* = 20) 0.19 (SD 0.18), Control (*n* = 26) 0.48 (SD 0.29); OR 5.29, *p* = 0.05	**1**	**1**
Barry (2022) [68]	Parenting skills	Community centre	Education + PS High dose Long term f/u	Group-based educational intervention providing blocks of weekly group sessions (90–150 min duration) over a period spanning 3 to 5 years	**Externalising behaviours**	Intervention (Los Angeles) OR 0.38 (95% CI 0.17 to 0.84), *p* ≤ 0.05	**1**	**1**
Dawson-McClure (2014) [79]^a^	Parenting skills	School + home-based	Education + PS High dose Long term f/u	13 × weekly (2 h) sessions for parents and concurrent sessions for children. Education included flyers and brief information sessions at school events. Delivered in person and by phone to parents. Designed to serve culturally diverse communities	**Parent involvement (parent rated)**	Intervention Estimate 0.78 (SE 1.55), d = 0.38	**1**	**1**
El-Mohandes (2003) [81]	Parenting skills	Home- based + community centres	Education + PS High dose Long term f/u	32 home visits and 16 play group sessions; weekly visits for first 5 months, followed by biweekly group sessions of developmental play groups and parent support groups (45 min). Monthly support calls, total duration 1 year	**Number of well infant visits at 12 months**	(Total n = 167) Intervention 3.51; Control 2.68, p = 0.0098	**1**	**1**
Fiks (2017) [86]	Parenting skills	Home- based	Education + PS High dose Long term f/u	2 educational sessions delivered in-person (1 prenatal and 1 at age 4 months), total duration 11 months (2 months prenatal and 9 months postnatal). Peer to peer Facebook group during intervention. Based on social cognitive theory	**Infant feeding behaviours: Total score**	Intervention 40.7; Control 38.2, ES = 0.45 (95% CI 0.01 to 0.92)	**1**	**1**
Hesselink (2012) [90]	Parenting skills	Community centres & home-based	Education + PS High dose Long term f/u	Antenatal education and parenting program involving 8 group classes (2 h each)—seven before and 1 after delivery, and 2 home visits (1 h each) after delivery. Quasi-experimental study	**SIDS prevention behaviour**	β = -0.024 (95% CI -2.9 to 2.4); favours control	**0**	**NS**
Jensen (2021) [95]	Parenting skills	Home-based	Education only High dose Long term f/u	Approximately 14 × 1 h home visits over a 9-month period. Followed an educational curriculum, included active play sessions with live feedback and linkage to government support service	**Harsh discipline**	‘Difference in difference’ 0.74 (95% CI 0.66 to 0.84), p < 0.001; favours intervention	**1**	**1**
Kasari (2014) [96]	Parenting skills	Home- based	Education only High dose Medium f/u	Individualized caregiver-mediated intervention with caregivers coached in the treatment model with their child. 2 x (1 h session) weekly sessions; duration 12 weeks (24 sessions, 24 h). Written material in participants native language	**Parent–child interaction: Total time in joint engagement**	Cohen’s f = 0.21 (“moderate treatment effect”)	**1**	**1**
Luten-bacher (2018) [101]	Parenting skills	Community centre + home-based	Education + PS Moderate dose Long term f/u	The Maternal Infant Health Outreach Worker program. Monthly individual home visits (1 h) and periodic group gatherings. Bilingual	**Breast-feeding duration (weeks)**	Intervention (*n* = 76) median 28.0 (IQR 12–28); control (*n* = 70) median 28.0 (IQR 12–28); *p* = 0.76	** < > **	**NS**
Mc Gilloway (2014) [107]a	Parenting skills	Community centre	Education + PS High dose Long term f/u	Incredible Years Basic parent program. 14 (2 h) sessions delivered over 12–14 week period, Education provided in groups using role plays and video material. Intervention culturally tailored, based on social cognitive theory	**Child problem behavior**	Mean difference 2.0 (95% CI 1.1 to 3.0), ES = 1.07	**1**	**1**
Pitchik (2021) [112]	Parenting skills	Community centre + home based	Education + PS High dose Long term f/u	2 intervention arms: 18 × 45–60 min Group sessions (with 3–6 women/caregivers); or 9 × group sessions alternated with 9 × 20–25 min home visits. The material covered was equivalent across the delivery mechanisms, duration 9 months	**Stimulating caregiving practices**	Group 4.22 (95% CI 3.97 to 4.47); combined 4.77 (4.60 to 4.96); control 3.24 (3.05 to 3.39); in favour of intervention	**1**	**1**
Segal-Isaacson (2006) [121]	Diet	Community centres	Education + skills training High dose Long term f/u	Nutrition education and coping skills/stress management sessions. Phase 1- high intensity received group sessions of therapist guided exercises. Phase 2—high intensity received behavioural exercises led by therapist plus expert advice from relevant professionals (nutritionist, exercise trainer or pharmacist). 10 group sessions and 6 behavioural exercises	Triglycerides	Group 1 (*n* = 97) 188 (SD = 103), group 3 (*n* = 79) 178 (SD = 96); *d* = *0.10 (95% CI -0.20 to 0.40)*	1	NS
Steptoe (2003) [125]^a^	Diet	Health clinics (primary care)	Education only Moderate dose Long term f/u	Individualised behavioural dietary counselling intervention targeted increasing intake of fruit and vegetables. 15-min consultation followed by another 15-min consultation after 2 weeks. Delivered individually face-to-face. Time matched with nutrition education counselling. Behavioral counselling integrated social learning theory and the stage of change model	**N**^**o**^** of portions of fruit/vegetables per day** Plasma β-carotene	Adjusted difference in change 0.89 (95% CI 0.25 to 1.54) Adjusted difference in change 0.18 (95% CI 0.02 to 0.37)	**1** 1	**1** 1
Zoellner (2016) [26]^a^	Diet	Community centre & home-based	Education + skills in self-monitoring High dose Medium f/u	SIPsmartER intervention: 3 small-group classes (90-120 min) (delivered in week 1, week 6 and week 17) + 1 live teach back call (avg of 18.6 min duration) + 11 interactive voice response calls (weekly for the first 3 weeks and then bi-weekly for the rest of the intervention) (avg 6.9 min duration of each call). Group classes delivered face-to-face. Culturally sensitive, integrated Theory of Planned Behaviour	**Sugar sweetened beverage consumption** Blood Glucose	Relative effect between cond-itions -14 (95% CI = -23 to -6) Relative effect between cond-itions -0.8 (95% CI -3.6 to 2.0)	**1** 1	**1** NS
Avila (1994) [37]	Diet & exercise	Community health clinics	Education + exercise Moderate dose Medium f/u	Weight reduction/exercise classes including 25-min exercise (stretching and walking) component with nutritional education, self-change behavioural modification strategies, buddy system and an exercise component. 1 h per week for 8 weeks. Bilingually delivered	**Exercise fre-quency (days/wk)** BMI	Intervention (*n* = 21) 3 (SD 2.6), control (*n* = 18) 1 (SD 2) Intervention 28.7 (SD 2.2) Control 32.0 (SD 2.27)	**1** 1	**1** 1
Baranowski (1990) [67]	Diet & exercise	Community centre or school	Education + counselling + exercise High dose Short term f/u	Program to improve diet and increase aerobic activity. Sessions involved education, behavioural counselling, food/activity records, goal setting, problem solving and aerobic activity. Intervention involved 1 × 90-min education and 2 fitness sessions per week for 14 weeks	**Per week energy expenditure** Resting pulse rate	Intervention (*n* = 50) 247 (SD 46.6); Control (*n* = 48) 248 (SD 29.4); d = -0.03 *(95% CI -0.42 to 0.37)* NS	**0** -	**NS** NS
Befort (2016) [69]	Diet & exercise	Community cancer centres	Education + PS High dose Long term f/u	Education program for breast cancer survivors Phase 2 (maintenance intervention) involving 25 biweekly conference call sessions. (Phase 1 included 25 weekly 60-min conference call sessions)	Weight change	Phone counselling (n = 85) 3.3 (SD 4.8); newsletter (n = 83) 4.9 (SD 4.9) *d* = *-0.33* (95% CI -0.63 to -0.03); favours phone counselling intervention	**1**	**1**
Brooking (2012) [53]	Diet & exercise (diabetes prevention)	Community centre	Education + PS + food High dose Long term f/u	Involved group and individual education sessions, written resources, cooking demonstrations and shopping tours. Weekly face to face contact with both group and individual. Three 8-week phases	Weight (kg)	Intervention (n = 20) 100.6 (SD 20.4); Control (n = 21) 97.7 (SD 20.01); *d* = *0.14 (95% CI -0.47 to 0.76);* favours control	**0**	**0**
Staten (2004) [56]	Diet & physical activity	Community centres	Education only High dose Long term f/u	Arm 1 – 1:1 counselling to increase fruit and vegetable consumption and physical activity, referral to education classes. Arm 2—counselling and health education plus education classes and a monthly newsletter. Arm 3—counselling, health education and community health worker support. Bilingual, based on social cognitive theory	**Physical activity levels > / = 150 min/week** High blood pressure	Intervention (arm 2, n = 70) % difference 2.6%, control (n = 73) % difference 0% Intervention (arm 2): 11.4% difference, control 11%	**1** 1	**NS** NS
King (2013) [38]	Physical activity	Community centres	Education + pedometer Moderate dose Medium f/u	4 × monthly virtual advisor sessions accessed on a computer, average 7 min each. Individually tailored walking program, physical activity education, personalised feedback, problem solving & goal setting. Culturally and linguistically tailored, bilingual intervention	**Increase in walking**	Between group difference 226.7 (95% CI 107.0 to 346.4), F(1,38) = 13.6, p = 0.0008, ES = 1.2	**1**	**1**
Reijneveld (2003) [114]	Physical activity	Community-based	Education + exercise High dose Short term f/u	8 × 2-h health education sessions offered by a peer educator. Each session ended with a group exercise session	**Physical activity** (low score = better)	Intervention (*n* = 54) 9.87; control (*n *= 38) 9.26; Difference -0.12 (95%CI -0.67 to 0.29) ES 0.04	**0**	**NS**
Alias (2021) [62]	Healthy lifestyle	Primary care clinics, community	Education + PS High dose, Long term f/u	12 × 2-h weekly sessions for groups of 15 people. 9 delivered in primary care centre; 3 involved local outings to public spaces (for physical activity/shopping/social activities)	**Social participation**	Between group data not reported. Raw data show results in favour of control group	**0**	**NS**
Fernandez-Jimenez (2020) [85]	Healthy lifestyle	Community or home- based	Education ± activity monitor High dose Long term f/u	Individual intervention 1: 8–12 counselling sessions with a lifestyle coach. Held every 3–4 weeks, lasting 45 min for first 8 months, 4 complimentary sessions offered over the following 4 months. Also provided with activity monitoring device. Group intervention 2: monthly group meetings for 12 months, 45 min each	**Change in a composite health score**	Group intervention: mean difference 0.00 (95% CI -0.50 to 0.49)	** < > **	**NS**
Hovell (2008) [29]	Healthy lifestyle	Community centre	Education + exercise High dose	Aerobic dance intervention (vigorous low impact aerobic dance sessions) plus 30 min exercise/diet education. 3 sessions per week (each 90 min) over 6 months. Culturally tailored and bilingual, developed for low literacy	**Moderate exer-cise** (min/2 wk) Relative VO_2max_	B = -0.184 (95% CI -0.87 to 0.497) *p* = 0.596; favours control B = 2.533 (95% CI 1.10 to 3.97), p < 0.001	**0** 1	**NS** 1
Keyserling (2008) [30]	Healthy lifestyle	Community health centre & home-based	Education + PS High dose Long term f/u	Lifestyle intervention to improve physical activity and diet. 2 individual counselling sessions, 3 × 90-min group sessions and 3 phone calls from a peer counsellor over 6 months, followed by a 6-month maintenance phase with 1 individual counselling session and 7 monthly peer counsellor calls. Reinforcement mailings of pamphlet & 2 postcards	**Moderate intensity physical activity** (mins/day)	Difference between means 1.5 (95% CI -1.6 to 4.6)	**1**	**NS**
Khare (2012) [27]	Healthy lifestyle	Community centre & home-based	Education only High dose Long term f/u	Minimum intervention—received CVD risk factor screening and educational materials. Enhanced intervention—also received a 12-week lifestyle change (nutrition and physical activity) intervention: 90-min weekly sessions for 12 weeks. Bilingual, based on social Cognitive Theory and Transtheoretical Model	**All intensity physical activity** (hours/week) BMI	MI (*n* = 280) 9.2 (SD 6.0); EI (*n* = 225) 9.7 (SD 6.6), *d* = *0.08 (95% CI -0.10 to 0.26)* MI (*n* = 280) 31.5 (SD 7.6); EI (*n* = 225) 31.8 (SD 7.7), *d* = *0.04 (95% CI -0.14 to 0.21)*	**1** 1	**NS** NS
Khare (2014) [28]	Healthy lifestyle	Community centre & home-based	Education only High dose Long term f/u	Minimum intervention—received CVD risk factor screening and educational materials. Enhanced intervention—also received a 12-week lifestyle change (nutrition and physical activity) intervention: 90-min weekly sessions for 12 weeks. Bilingual, based on social Cognitive Theory and Transtheoretical Model	**All intensity physical activity** BMI	MI (*n* = 37) 10.0 (SD 5.61); EI (*n* = 30) 8.48 (SD 5.73), *d* = *0.27 (96% CI -0.22 to 0.75)* MI (*n* = 37) 32.03 (SD 8.06); EI (*n* = 30) 30.22 (SD 5.57), *d* = *0.26 (95% CI -0.23 to 0.74)*	**1** 1	**NS** NS
Kim (2021) [98]	Healthy lifestyle	Community centre	Education + exercise Moderate dose Short term f/u	8 week group-based intervention addressing nutrition, exercise, stress management psychological wellbeing and cognitive health. Involved education and physical activity components plus recommended daily exercise (> 10,000 steps or > 30 min mod exercise per day)	**Health promot-ing behaviour** % body fat	d = 1.27, p < 0.001; results favour intervention d = 0.53, *p* = 0.62; results equivocal for both groups	**1** (< >)	**1** (NS)
Parra-Medina (2011) [31]	Healthy lifestyle	Home- based	Education only High dose Long term f/u	Standard care plus 12 motivational, ethnically tailored newsletters over 1 year, an in-depth introductory telephone call, & up to 14 brief, motivationally tailored telephone counselling calls from research staff over 1 year. Print materials for less than 8^th^ grade reading level, based on transtheoretical model and social cognitive theory	**Improvement in moderate-to-vigorous physical activity**	(*n* = 142) Intervention 30.7%, control 44.8%; OR 0.63 (95% CI 0.24 to 1.68); favours control	**0**	**0**
Polomoff (2022) [113]	Healthy lifestyle	Community centres	Education + PS + medication management High dose Long term f/u	A bilingual, trauma-informed, cardiometabolic education intervention to decrease diabetes risk. 2 intervention arms: Eat,walk sleep (EWS) (or EWS + 3 or more MTM (medication therapy management) sessions. EWS involved 3 individual sessions and 24 group sessions over a 12-month period	**Medication forgetting**	Results in favour of intervention but between-group differences not significant	**1**	**NS**
Seguin-Fowler (2020) [122]	Healthy lifestyle	Community-based	Education + PS + exercise, High dose Medium term f/u	24 weeks of hour-long, twice weekly classes held in community-based locations. Sessions included strength training, aerobic exercise and health related education, civic engagement activities and out of class assignments	**Moderate and vigorous physical activity** Total cholesterol	Intervention: 41.5% improved, control: 21.5% improved (*p* = 0.008) 2.8% difference, *p* = 0.66); favours intervention	**1** 1	**1** NS
Saleh (2018) [119]	Healthy lifestyle	Community centre & home-based	Education only High dose Long term f/u	Weekly short message service over 2 years. Messages included medical information & reminders of appointments. Information included hypertension and diabetes guidelines for management, dietary habits, body weight, smoking	Blood pressure controlled at post-test	Intervention (*n* = 426) 63.6%; control (*n* = 362) 58.4%; *OR* = *0.80 (95% CI 0.60 to 1.07)*	1	NS
Hayashi (2010) [40]	Healthy lifestyle	Community health centres	Education only Moderate dose Long term f/u	WISEWOMAN Program: 3 sessions (at 1, 2, 6 months post enrolment). Initial session of 40–70 min, 3 lifestyle intervention sessions lasted 30–45 min. Delivered face-to-face. Bilingual and bicultural Intervention, outcome measures selected based on transtheoretical model	**Improvement in eating habits** Total cholesterol > 240 mg/dL	Intervention (*n* = 433) 71%; Control (*n* = 466) 48%; RR 3.3, *p* < 0.001; favours intervention Intervention 200.3; control 199.3, *p* = 0.906; favours control	**1** 0	**1** NS
Suhadi (2018) [57]	Healthy lifestyle	Community centres	Education only Moderate dose Long term f/u	Oral presentations and discussion of topics such as hypertension, hyperlipidaemia and diabetes. Participants were handed posters, activity manuals and 4 booklets with education material. 4 sessions of 90-min each done consecutively every 1–2 months	BMI	Intervention (*n* = 82) 24.1 (SD 4.5); control (*n* = 108) 24.0 (SD 4.4); *d* = *0.02 (95% CI -0.26 to 0.31)*	0	NS
Fitzgibbon (1996) [87]	Healthy lifestyle (diet/breast health)	Community centre	Education only High dose Short term f/u	12 weeks × 1-h classes. Culture specific family-based dietary intervention to reduce cancer risk among low-literacy, low-income Hispanics by reducing fat intake, increasing fibre intake, increasing nutrition knowledge and increasing parental support for healthy eating	**Saturated fat intake** Blood pressure	Intervention (*n* = 18) 11.2 (SD 4.0), control (*n* = 18) 13.6 (3.1) NS	**1** -	**NS** NS
Bray (2013) [71]^a^	Diabetes self-man-agement	Health clinics	Education only High dose Long term f/u	Point of care diabetes care management involved education, self-management coaching and medication adjustment. 1:1 face to face sessions. Patients seen an average of 4 times over 12 months by a nurse, pharmacist, or dietitian care manager for 30–60 min, seen every 3–6 months by a care manager for an additional 2 years. Quasi-experimental study	Haemoglobin A1C	Intervention (*n* = 368) 7.4 (SD 1.9); Control (*n* = 359) 7.8 (SD 2.0), d = -0.21	1	1
Brown (2013) [73]	Diabetes self-man-agement	Community centre	Education + PS High dose Long term f/u	Culturally tailored diabetes self-management education including educational videos and group activities. Conducted near participants home, required to partner with a relative/friend. 1 year duration with 52 contact hours. 26 educational and group support sessions (each 2 h)	Haemoglobin A1C	Females (*n* = 70): Intervention 10.8 (SD 2.5), Control 11.5 (SD 3.0); NS	1	NS
Andrews (2016) [64] + a	Smoking cessation	Community centres + home-based	Education + PS + Nicotine replacement Moderate dose Long term f/u	6 × weekly group sessions. Community health workers provided 1:1 contact (× 16) to reinforce educational content and behavioural strategies from the group sessions & provide social/psychological support. 24-weeks duration	**7-day point prevalence abstinence**	OR = 0.44 (95% CI 0.18 to 1.07), favours intervention	**1**	**NS**
Berman (1995) [70]	Smoking cessation	School- based	Education + PS High dose Long term f/u	Smoking cessation group class – seven sessions, 1.5 h each. Received tailored support letters and brief tailored smoking cessation booster messages at end of 3- and 6-month interviews. Quasi-experimental study	**Continuous abstinence**	(Total *n* = 132), Intervention 6.4%; Control 7.3%; χ^2=^0.042; RR = 0.88; favours control	**0**	**NS**
Brooks (2018) [72]	Smoking cessation	Home- based	Education + MI Moderate dose Long term f/u	Up to 9 education sessions from a Tobacco Treatment Advocate over 6 months, Delivered in person (at home). Involved motivational interviewing and cognitive behavioural strategies and cessation counselling. Also offered community resources + educational materials. Considered racial and linguistic diversity	**30-day point prevalence abstinence**	Adjusted OR 2.98 (95% CI 1.56 to 3.94)	**1**	**1**
Curry (2003) [77]^a^	Smoking cessation	Outpatient paediatric health clinics	Education + MI Moderate dose Long term f/u	Pediatric setting-based smoking cessation intervention where women received a motivational message from the child's clinician, a guide to quitting smoking and a 10-min motivational interview with a nurse or study interventionist followed by up to 3 outreach telephone counselling calls over 3 months	**7-day point prevalence abstinence**	OR = 2.12 (95% CI 0.96 to 4.66)	**0**	**NS**
Gielen (1997) [89]	Smoking cessation	Health clinic	Education + PS Low dose Long term f/u	Individual skills instruction and counselling by a peer health counsellor. 1:1 (15 min) counselling session, clinic reinforcement and support including two letters of encouragement mailed 1–2 weeks after first visit	**Smoking status: quit rate** (self-report & saliva cotinine test)	Intervention (*n* = 193) 6.2%; control (*n* = 198) 5.6%; *OR* = *0.89 (95% CI 0.38 to 2.06)*	**1**	**NS**
Hooper (2017) [91]	Smoking cessation	Research clinic	Education + CBT + Nicotine patches High dose Long term f/u	Group based cognitive behavioural therapy for smoking and health, self-motivation and goal setting with culturally specific education. 8 sessions: 4 during week 1, 2 during week 2 and 2 booster sessions weeks 3 and 4. Session duration 90–120 min	**7-day point prevalence abstinence** (biochemically verified)	Intervention (*n* = 168) 23.2; control (*n* = 174) 22.0; OR 1.21 (95% CI 0.71 to 2.04)	**1**	**NS**
McClure (2020) [105]	Smoking cessation	Home-based (telephone)	Education only Moderate dose Medium f/u	4–5 sessions of telephone counselling plus scripted educational content, mailed oral health promotion brochure, access to online (educational) information and oral health messaging in 16 text messages	**Meet brushing and flossing** **recommendation**	Adjusted OR 1.16 (0.96,1.41), *p* = 0.13; raw data in favour of intervention	**1**	**NS**
Simmons (2022) [123]	Smoking cessation	Home-based (via mail)	Education only High dose Long term f/u	Participants received a series of 11 booklets and 9 pamphlets over a 18 month period, and a 10 min phone call one week after randomisation	**7-day point prevalence smoking abstinence**	Abstinence rates: intervention 33.1%, control 24.3%; OR 1.54 (95% CI 1.18 to 2.02), *p* = 0.002	**1**	**1**
El-Mohandes (2010) [82]	Tobacco smoke exposure	Health clinics	Education + CBT/safety plan High dose Medium f/u	10 × behavioural counselling intervention sessions occurred in conjunction with prenatal and post-partum health checks. Based on behaviour change literature	**Environmental tobacco smoke exposure**	Intervention (*n* = 335) 53.9; control (*n* = 356) 68.2, Adjusted OR 0.50 (95% CI 0.35 to 0.71)	**1**	**1**
Emmons (2001) [83]^a^	Tobacco smoke exposure	Home- based	Education + MI Moderate dose Long term f/u	Motivational interview at client's home and 4 follow up telephone counselling calls over 6 months, quit magazines. Tailored for men and women and in English and Spanish, theory driven approach	**Nicotine level: TV room** (mg/m^3^)	Intervention (*n* = 150) 2.3; control (*n* = 141) 3.5, F(1235) = 5.04, p < 0.05	**1**	**1**
Byrd (2013) [32]^a^	Cancer screening	Community centres	Education only Low dose Medium f/u	Bilingual program delivered by a lay health worker: (i) full program included video and flip chart (educational information, games, and activities); (ii) program without video; (iii) program without flip chart. All received educational handouts, cards and 1 × face-to-face session	**Validated pap smear**	Intervention (full program: *n* = 151) 17.9%, Control (*n* = 152) 7.2%, *OR* = *0.35 (95% CI 0.17 to 0.75)*	**1**	**1**
Calderon-Mora (2020) [44]	Cancer screening	Community centre	Education + PS Low dose Medium f/u	Group program comprised of outreach, educational session, navigation services, and no cost cervical cancer testing. Used flipchart, message cards, action plan worksheet, resource sheet and informational handouts. Mean duration 90 min with 3–6 participants. Bilingual	**Self-reported cervical cancer screening**	Intervention (n = 150) 68.9%; control (n = 125) 77.6% ITT RR (adjusted) 0.95 (95% CI 0.80 to 1.13), p = 0.59	**0**	**NS**
Dooren-bos (2011) [43]^a^	Cancer screening	Home- based	Education only Low dose Long term f/u	Participants were mailed a calendar with cancer screening messages and screening service information	**Breast cancer screening mammogram**	Intervention 14.0%; control 13.6%; no effect, *OR* = *0.96 (95% CI 0.83 to 1.13)*	**1**	**NS**
Fitzgibbon (2004) [41]	Cancer screening	Community centre	Education only High dose Long term f/u	16 (90 min) sessions: once per week for 8 weeks, biweekly for 2 months and once monthly for 4 months; Education provided in groups led by a research nutritionist and a trained breast health educator; duration 8 months. Bilingual Intervention	**Breast self-examination frequency**	Intervention (n = 92) 45.7%; Control (n = 103) 22.3%; *OR* = *0.34 (0.18 to 0.63)*	**1**	**1**
Gathirua-Mwangi (2016) [33]	Cancer screening	Home- based	Education only Low dose Long term f/u	Two interventions compared with control group: mailed interactive DVD (10 min duration) and a tailored telephone counselling intervention (approximately 11 min duration). Both delivered similar messages related to importance of mammograms	**Mammography adherence rates**	DVD: OR = 1.64 (95% CI 0.80 to 3.39); Telephone: OR = 1.24 (95% CI 0.61 to 2.50)	**1**	**NS**
Kalichman (2000) [42]	Cancer screening	Community centre	Education only Low dose Medium f/u	Single session; 2.5 h duration; small group workshop; delivered in person. Intervention culturally tailored, based on social cognitive theory	**Performance of monthly breast self-examination**	Intervention (n = 15) 52%; control (n = 6) 25%; OR = 4.68 (95% CI 1.3–18.4)	**1**	**1**
Katz (2007) [34]	Cancer screening	Home- based	Education only Moderate dose Long term f/u	Lay health advisor education program. 3 home visits, follow up phone calls and tailored mailings after each visit. First visit 45–60 min, 2nd visit 2–3 weeks later 30–45 min, tailored phone calls/mailings in months 3–9, final visit 10–14 months	**Cervical cancer screening rates**	(n = 792) OR^a^ = 1.03 (95% CI 0.80 to 1.32)	**1**	**NS**
Kreuter (2005) [35]	Cancer screening & diet	Home based	Education only Moderate dose Long term f/u Home-based	6 women's health magazines promoting use of mammography for ages 40–65 and promoting fruit and vegetable intake for ages 18–39. Three intervention arms: behavioural construct tailoring, culturally relevant tailoring, or both. Culturally tailored	**Use of mammography**	Intervention (both) (n = 45) 75.6%; control (n = 55) 54.5%, *OR* = *0.39 (95% CI 0.16 to 0.92)*	**1**	**1**
Kreuter (2010) [45]	Cancer screening	Neighbour-hood & home-based	Education only Low dose Long term f/u	Narrative video comprised of stories from African American breast cancer survivors OR content equivalent information video. Delivered in a mobile research van in participants neighbourhood, follow up questionnaire administered by phone	**Use of mammography**	Narrative video (n = 107) 48.6%; Informational video (n = 115) 40.0%; *OR* = *0.71 (95% CI 0.41 to 1.20)*	**1**	**NS**
Ridgeway (2022) [116]	Cancer screening	Health clinics	Education only Low dose Short/Med f/u	2 intervention arms: The enhanced care group were provided with an educational brochure along with their results letter; the interpersonal group received follow-up telephone interaction and education (along with the educational brochure)	**Self-reported provider conversations:**	Between group difference in favour of intervention, p < 0.001	**1**	**1**
Valdez (2016) [36]	Cancer screening	Community health centre	Education only Low dose Medium f/u	One -time, low-literacy, interactive cervical cancer education program Education was individualised, self-paced via a multimedia kiosk (2 languages and age category options) involved 8 interactive education modules. Average duration 24 min (English) and 28 min (Spanish)	**Self-reported cervical cancer screening**	Intervention (*n* = 138) 51%; control (*n* = 344) 48%, p = 0.35 *OR* = *0.90 (95% CI 0.60 to 1.33)*	**1**	**NS**
Jacobson (1999) [93]	Vaccina-tions	Health clinic	Education only Low dose Short term f/u	Single session: education provided by a 1-page document given before a doctor’s appointment. Designed for low literacy levels	**Discussion of vaccination with physician**	Intervention (*n* = 221) 39.4%; control (*n* = 212) 9.9%; RR 3.97 (95% CI 2.71 to 5.83)	**1**	**1**
Falbe (2015) [84]	Family health	Health clinics & home-based	Education only High dose Short term f/u	Family centred; culturally tailored group intervention. Covered topics such as parenting, screen time, healthy beverages, physical activity and stress due to immigration. 10-week, biweekly group sessions lasting 2 h each. Two between-session phone calls	BMI	Adjusted difference in change -0.78 (95%CI -1.28 to -0.27), p = 0.004	1	1
Phillips (2014) [111]^a^	Ear health (children)	Home-based	Education Moderate dose Long term f/u	Seven ear health multi-media messages (over 6 weeks) in local Indigenous language, accompanied by personalised ear health text messages in English, with prompts to visit the clinic for the children's health check-ups. Included short, caricature animation videos of Indigenous role models	**Clinic attendance**	Mean difference -0.1 (95% CI -1.1 to 0.9)	**0**	**0**
Janicke (2008) [94]	Weight loss (children)	Community centre	Education + Pedometer High dose Long term f/u	Behavioural family-based OR parent-only diet and weight loss educational intervention. In both groups families and group leaders set daily dietary goals at end of each group sessions, increased physical activity promoted through pedometer. Weekly group sessions for first 8 weeks, biweekly for the next 8 weeks, sessions lasted 90 min	**Change in children's standardized BMI**	Intervention (family) (*n* = 24) mean change -0.115 (SD 0.22); control (n = 21) mean change 0.022 (SD 0.17), *p* < 0.05	1	1
Smith (2021) [124]	Weight loss (children)	Health clinic + home based	Education ± community services High dose Long term f/u	An individually tailored intervention designed to pre-empt excess weight gain by improving parenting skills. Delivered for 6 months in clinic, at home and in the community with a dose target of 26–50 h of support. Support included face to face and telephone coaching and connection to community-based services	**Health routines** BMI	d = 0.33; β = 0.16 (95% CI 0.009 to 0.291), *p* = 0.037; favours intervention No between group differences: d = − 0.01, *p* = 0.96	**1** ** < > **	**1** 0
Kelly (1994) [97]	Sexual health	Health clinics	Education + PS Moderate dose Medium f/u	Group sessions focusing on risk education, skills training in condom use, sexual assertiveness, problem solving, and risk trigger self-management and peer support for change efforts. 5 x (90 min) 4- weekly group sessions and a 1-month group follow up	**Frequency of unprotected sexual intercourse**	Intervention 11.7 (SD 22.8); control 15.0 (SD 26.4); *d* = *-0.13 (95% CI -0.42 to 0.15)*	**1**	**NS**
Kulathinal (2019) [100]	Sexual health	Community education + home based	Education + contraceptives Variable dose Medium f/u	Involved a mobile helpline, mid-media activities (including street art, theatre), personal contact from village health workers and distribution of contraceptives. Total duration of intervention period 12 months. Questionnaire tailored for low literacy	**Uses contraception**	Intervention 42.9%; control 40.8%; OR 3.207 (95% CI 3.03–3.39); favours intervention	**1**	**1**
Miller (2013) [108]	Sexual health	Home- based	Education only Low dose Long term f/u	Arm 1: telephone assessment of barriers to adherence and tailored counselling. Arm 2:as arm 1, plus mailing of a tailored information brochure. Arm 3 – standard care (telephone assessment only)	**Adherence rates to initial colposcopy**	Intervention 75.4%; control 65.75%, p = 0.23, *OR* = *0.94 (95% CI 0.47 to 1.87)*	**1**	**NS**
Robinson (2002) [117]	Sexual health	Community centre	Education + PS High dose Long term f/u	Education of HIV and sexually transmitted disease prevention strategies plus comprehensive sexuality education. Sessions were multimedia and multimethod including peer panels, storytelling, exercises, small group support and discussions. 2-day program	**Frequency of unprotected intercourse**	f = 0.339, df = 1,101; p = 0.562; (direction of effect unclear)	**-**	**NS**
Santa Maria (2021) [120]	Sexual health	Community-based	Education only Moderate dose Medium f/u	Parents received a 1:1 individual 45-min information session, were provided with an education manual and received 2 booster phone calls	**HPV vaccine completion**	Study concluded no difference between the groups. No raw data available	**-**	**NS**
Kim (2014) [54]	Hyper-tension	Community centre & home-based	Education + monitoring device High dose Long term f/u	6 × weekly, 2-h education sessions (including overview of high blood pressure management guidelines, complications, healthy diet, exercise, medications, problem solving skills); participants given a blood pressure monitoring machine and asked to take blood pressure twice a day; monthly telephone counselling for 12 months	Blood pressure control rates	Intervention (n = 184) 54.3%; control (n = 185) 53.0%, OR = 0.95 (95% CI 0.628 to 1.42)	1	NS
Kisioglu (2004) [55]	Hyper-tension & obesity	Community centre & home-based	Education only Low dose Long term f/u	Group sessions of 5. All women in the intervention group received health training support from an expert and a leaflet. No limit applied to session length. (Daily exercise advised)	Blood pressure (optimum)	Intervention 54%; control 50%, p = 0.31, *OR* = *0.85 (95% CI 0.58 to 1.26)*	1	NS
Martin (2011) [104]	Hyper-tension	Home- based	Education only Moderate dose Long term f/u	Medication adherence intervention via computer; a community health advisor; and telephone contact. Involved 4 home visits over a 6-month period with telephone contact at 2 weeks post session after each home visit. Program used 50 videos ranging 10–60 secs	**Pill count** (adherence to medication)	N = 338, Intervention 51%, control 49%, p = 0.67, *RR* = *1.04*	**1**	**NS**
Almabadi (2021) [60]	Dental health	Dental health clinic	Education + oral health care High dose Long term f/u	Program provided information regarding oral hygiene procedures, smoking and alcohol cessation, healthy diet	**Vegetable consumption** Sites with PPD > 5 mm	Greater improvement in treatment group at 12 months Equivocal results both groups	**1** < >	**1** NS
Cibulka (2011) [76]	Dental health	Hospital health clinic	Education + dental supplies Low dose Medium f/u	1:1 education session with dental nurse practitioner. Five-minute section of a digital video disc and scheduling of an oral health appointment	**Attend dental check up**	Intervention 56.9%; Control 32.9%; Pearson’s χ^2^ = 7.544, df = 1, p = 0.006, *OR* = *0.37 (95% CI 0.19 to 0.73)*	**1**	**1**
Dela Cruz (2012) [80]	Dental health	Home- based	Education only Low dose Long term f/u Home-based	Post card mailing about benefits of dental health care. 1 postcard for group 1; 3 postcards for group 2 over 1 year	**Preventive dental service utilisation rates**	No significant between group differences (61% vs 62% vs 61%), *RR* = *1.02 (group 2 vs control)*	**1**	**NS**
Krieger (2005) [99]	Asthma	Home- based	Education + household equipment High dose Long term f/u	Involved education, social support, resources to reduce exposure (allergy control pillow, mattress encasements, vacuums, cleaning kits, referral to smoking cessation counselling, roach bait, rodent traps), skin prick allergy testing. 7 visits and resources over 12 months. Delivered in English, Spanish & Vietnamese	**Behaviour summary score**	High intensity (n = 104) 8.0, low intensity (n = 104) 6.4, GEE coefficient (group x time interaction) 0.41 (95% CI -0.13 to 0.95), p = 0.11	**1**	**NS**
Damush (2003) [78]	Low back pain	Health clinic & home-based	Education only Moderate dose Long term f/u	Self-management program involving 3 face-to-face group sessions (once per week), class handouts with written education materials, audio cassettes if missed session, phone follow up, physician letters of support after each session	**Total physical activity**	Intervention 178.1 (SD 149.3); control 152.5 (SD 159.3); effect estimate 42.0 (95% CI 0.63 to 38.87), *d* = *0.14 (95% CI -0.19 to 0.48)*	**1**	**NS**
Cahill (2018) [74]	Healthy pregnancy	Home- based	Education only High dose Long term f/u	Home based lifestyle weight management intervention. Included goal setting, regular self-assessment of weight, education about positive eating and physical activity behaviours, observational learning through role play and environmental changes in the home. 10 biweekly home visits lasting 1 h through duration of pregnancy	% Whose gestational weight gain exceeded guidelines	Intervention (*n* = 133) 36.1%; Control (*n* = 134) 45.9%, *p* = 0.11	1	NS
Hillemeier (2008) [39]	Healthy pregnancy	Community centre	Education + PS Moderate dose Medium f/u	Strong Healthy Women program: 6 × biweekly group sessions; duration 12 weeks. Designed for low literacy, based on social cognitive model	**Physical activity** BMI	OR 1.867, *p* = 0.019; favours intervention Intervention effect -0.036, *p* = 0.809	**1** 1	**1** NS
Hunt (1976) [92]	Healthy pregnancy	Health clinics	Education + vitamins Moderate dose Medium f/u	5 nutrition education sessions. Women taught how to plan nutritious meals, and buy, store and prepare these foods. Also given vitamin and mineral capsules. Delivered in native tongue	**Dietary iron** (% of recommended daily intake) Serum folic acid deficiency	Intervention 58%, control 51% Intervention group 10% deficient, control group 15%, *p* < 0.05	**1** **1**	**NS** **1**
Reisine (2012) [115]	Healthy pregnancy	Community health centre	Education only Moderate dose Long term f/up	Arm 1—education alone, Arm 2—education and a 1-h nutrition group session at 9 months and 6 weeks postpartum. Nutrition sessions were small group based educational materials at 9-month prenatal visit	**Mutans levels**	Decrease in mutans over time did not differ by group F(3,110) = 2.6, p > 0.05; favours educational alone	**1**	**NS**
Acharya (2015) [59]^a^	Pregnancy & newborn health	Community education ± group meetings	Education only Variable dose Long term f/u	Large scale, 3-year intervention via district-level campaigns. Included advocacy (delivery of health messages during community events) & mass media messaging (posters, vehicle branding, street theatre & newsletters). High intensity intervention also involved community field workers in village health & sanitation committees, home visits to pregnant women & encouragement to attend monthly group meetings	**Healthy delivery behaviours** (Composite score)	OR = 1.507 (95% CI 1.248 to 1.818); favours intervention	**1**	**1**
Hoodbhoy (2021) [128]	Pregnancy & newborn health	Community + home based	Education only Low dose Long term f/u	The community engagement strategy had 2 components—a 45-min community-based; and 2 × interactive sessions were delivered to pregnant women and their families in their own homes	**Birth preparedness**	Intervention 43.87%, Control 29.72%, OR 1.74 (95% CI 0.64 to 4.73), p = 0.278	**1**	**NS**
Manandhar (2004) [103]	Pregnancy & newborn health	Community centre	Education + PS Variable dose Long term f/u	Community-based participatory intervention to improve childbirth and care behaviours. A female facilitator convened nine women’s group meetings every month to identify and prioritise peri-natal problems and formulate strategies to address them. 12-month duration	**Any iron and folic acid supplements**	Intervention 49%; control 30%; adjusted OR 1.99 (95%CI 1.14 to 3.46)	**1**	**1**
Pandey (2007) [110]	Pregnancy & newborn health	Community education ± group meetings	Education only Variable dose Long term f/u	Two to 3 public meetings were held in each village cluster to disseminate information on entitled health & education services. Education provided in groups using role plays and video material and distribution of posters and leaflets	**Visit by nurse/midwife**	Intervention 63%; control 61%, p = 0.15, *RR* = *1.03*	**1**	**NS**
Abiyu (2020) [58]	Newborn health	Community centre + home based	Education only High dose Long term f/u	Community based leaders delivered intervention involving 9 group sessions and 9 home visits over a 9-month period. Involved talks, group discussions, group work exercises, demonstrations, role plays, story- telling, simulation, case studies and problem-solving	**Minimum dietary adversity**	RR 3 (95% CI 1.34 to 7.39); favours intervention	**1**	**1**
Alvarenga (2020) [63]	Newborn health	Health centres	Education only High dose Long term f/u	Each of the 8 visits had 2 parts: part 1- the mother was video-recorded playing with the baby, part 2—the mother and intervener watch selected scenes and discuss ways to facilitate development	**Describes toy/activity**	Intervention 8.31 (95% CI 7 to 94) vs Control 4.81 (95% CI 4 to 84); favours intervention, not significant	**1**	**0**
Childs (1997) [75]	Newborn health	Home- based	Education only High dose Long term f/u	Dietary health education program—sessions delivered face to face plus educational resources (video and leaflets). Multiple sessions over a period of 18 months	**Breast feeding at 9 months** Haemoglobin (% with anaemia)	Intervention 6% (SD = 3); control 6% (SD = 2) Intervention 28%; control 27%; no significant difference	** < > ** 0	**NS** NS
McConnell (2016) [106]	Newborn health	Home– based	Education only Low dose Short term f/u	Arm 1—Early postnatal care three days after delivery provided in person with a community health worker using a checklist. Arm 2—Care provided by phone with a community health worker checklist. 1 session for each plus follow up phone call	**Postnatal health practices (composite score)**	Intervention arm 2: mean 7.2, control mean 6.6, p = 0.06	**1**	**NS**
Murthy (2019) [109]	Newborn health	Home based	Education only High dose Long term f/u	Voice messages delivered 2 × per week throughout pregnancy and until infant turned 1 year of age with a cluster of one message per day immediately postpartum for 7 days for a total of 145 voice messages	**Infant immunization status**	OR 1.51 (95%CI 1.14 to 2.06), *p* = 0.005	**1**	**1**
Ryser (2004) [118]	Newborn health	Health clinics	Education + counselling Moderate dose Medium f/u	4 sessions provided in conjunction with pre-natal visits. Involved educational videotapes, reading material and provision of counselling. Designed to address common breastfeeding barriers. Bilingual availability	**Initiation of breastfeeding**	Intervention 60.9%, control 14.8%; χ^2^(1, n = 50) = 9.52, p < 0.01, *OR* = *0.38 (95% CI 0.10 to 1.44)*	**1**	**1**
Wiggins (2005) [126]	Newborn health	Community centre & home-based	Education only High dose Long term f/u	Community group support intervention for mothers with children less than 5 years. Standard package included drop-in sessions, home visiting (monthly visits for 1 year) and/or telephone support	**Maternal smoking**	RR 0.86 (95% CI 0.62 to 1.19); favours intervention	**1**	**NS**

^a^ ‘Unclear’ risk of bias. (All other studies ‘high’ risk of bias)

^b^ Standardised metric – assigned according to Cochrane vote count methods: 1 = point estimate in favour of intervention; 0 = point estimate in favour of control group; <  >  = effect of intervention equivocal (intervention = control);—unable to determine direction of effect

NS, not significant (results reported as not statistically significant)

^c^ VC_C_ = conservative vote count – assigned according to whether individual studies concluded effectiveness

() indicate biomarker outcomes

^d^ See Appendix 3 for dose classification, follow-up classification: < 3 months = short term follow-up, 3–6 months = medium term follow-up, > 6 months = long term follow-up

Abbreviations: *PS* peer support,  *f/u* follow-up, *OR* odds ratio, *RR* risk ratio, *CI* confidence interval, *SD* standard deviation, *BMI* body mass index, *CVD* cardio-vascular disease, *HIV* human immunodeficiency virus, *HPV* human papilloma virus, *PPD* probing pocket depth

### Secondary objective: Characteristics of effective interventions

Narrative synthesis of the features of ‘effective’ versus ‘ineffective’ interventions was precluded by the high clinical and statistical heterogeneity of the included studies. We have organised the studies according to the health focus of the intervention in Table 3. This table provides descriptions of the main characteristics of the interventions alongside indications of effectiveness in order to facilitate reader interpretations.

## Discussion

We aimed to (i) identify and synthesize evidence of the effectiveness of health-related educational interventions in adult disadvantaged populations, and (ii) summarise the characteristics of effective interventions. When studies were sufficiently homogenous to allow data pooling, meta-analyses revealed that health education interventions targeting socially disadvantaged populations produced positive behavioural effects that were small or negligible in magnitude. The certainty of evidence was low (at best). Our vote-count syntheses found a marked discrepancy in the proportion of effective interventions depending on the method applied to classify benefit (i.e., 85% versus 43% for behavioural outcomes and 83% versus 31% for biomarker outcomes). The evidence included in this review did not demonstrate consistent, positive impacts of educational interventions on health behaviours or biomarkers in socio-economically disadvantaged populations. We were unable to draw conclusions related to the common features of ‘effective’ interventions due to the high clinical and statistical heterogeneity of the included studies.

Meta-analysis of the six sufficiently homogenous studies aiming to increase physical activity showed no effect, but the four studies that were *not* included in the meta-analysis due to heterogenous outcomes reported significant improvements in the physical activity outcome compared to control interventions [37–40]. Of these four positive studies however, two had fewer than 50 participants [37, 38] and two had drop-out rates exceeding 48% [37, 39]. Thus, evidence suggests it is unlikely that educational interventions had changed physical activity in disadvantaged populations.

Educational interventions were shown to have a small, pooled effect (Hedges g = 0.3) on cancer screening rates, however certainty for this evidence was rated as ‘very low’. Five studies investigating cancer screening uptake were not included in this meta-analysis – two used varied outcomes (self-reported breast self-examination), [41, 42] two were low-dose, [42, 43] and two had comparison groups that were active interventions [44, 45]. From these studies, the interventions were effective for the two studies with breast self-examination outcomes, one of which analysed only 21 participants at follow-up. Based on the findings of the studies not included in the meta-analysis, the lack of evidence of benefit combined with the low quality of evidence reinforces that educational interventions to boost cancer-screening had, at best, small effects on cancer screening.

This review of evidence concerning the effectiveness of health-related educational interventions that target socio-economically disadvantaged populations is less encouraging than reviews of other health interventions in socio-economically disadvantaged groups. One review of mixed interventions for diabetes care [46] including novel providers’ roles, education and resources, found positive outcomes in 11 of the 17 included studies. The authors suggested that cultural tailoring, individualised components, multiple contacts (> 10), providing feedback, and involving community educators or lay people in delivery, were associated with better outcomes.

Our findings also show a stark contrast to the positive effect observed from health education interventions in non-disadvantaged socio-economic groups. Educational interventions designed to improve health-related behaviours such as oral health practices (15 studies), [47] foot self-care practices amongst diabetics (14 studies), [48] and cervical cancer screening rates (17 studies), [49] seem to provide mostly meaningful benefit. Education to promote self-management of hypertension demonstrated benefits on blood pressure outcomes in a systematic review of education programs that also targeted self-efficacy (14 studies) [50]. This contrast seems critically important because it raises the distinct possibility that educational interventions that are widely endorsed on the basis of their apparent effects, are often failing to meet the needs of the very people most likely to need them [51].

There are strengths and limitations of this work. We applied contemporary standards of transparency [52] and rigour, and reporting was in line with the PRISMA and PRISMA-E templates, and SWiM guidelines. We were unable to perform meta-analysis on a large majority of included studies due to heterogeneity. We synthesised data from these studies using two vote-counting methods: 1) studies were categorised as positive or negative based on direction of effect, regardless of effect size, and 2) studies were categorised as positive if the authors concluded the intervention was effective. The former method is recommended by Cochrane and does not consider statistical or clinical significance. Critically, neither approach provides estimates of the size of effects which is needed for policy or clinical decisions. The two synthesis methods provided very different results. Method 1 resulted in 83% of positive studies for behavioural outcomes and 75% for biomarkers, Method 2 resulted in 47% and 27% respectively. This inconsistency casts significant doubt over the usefulness of vote-counting approaches and means that we have very low certainty in our conclusions.

There may have been studies eligible for inclusion that were not identified by our database searches. For example, searching for specific conditions (e.g. diabetes) may have identified relevant studies *not* identified in our more general search for ‘health-related’ interventions; and studies that involved education as components of an intervention without explicit mention of this may have been missed. Citation chaining may also have identified further eligible studies. While not searching grey literature can contribute to an over-estimation of effectiveness (since null findings are less likely to be published in peer reviewed journals), this is unlikely to impact the findings of our review since most of the included studies concluded a lack of effect. Our evaluation of publication bias also suggests that this is not likely to be of major concern. Finally, it is important to acknowledge that we applied a very broad definition of socio-economic disadvantage when selecting studies for inclusion. While included studies most commonly involved participants with low income, types of disadvantage were also widely disparate (e.g., low educational attainment, living in rural areas, ethnic minority groups). Subgroup analyses of these factors was precluded due to study heterogeneity, such that it remains undetermined whether these varied types of disadvantage differentially impacted involvement in clinical trials or responsiveness to interventions. The impact of contextual factors associated with the economic classifications of the countries in which the study was conducted (e.g., lower middle income vs high income) is also unknown.

## Conclusions

This review highlights that health-related educational interventions tested to date have not consistently demonstrated positive impacts on health behaviours or biomarkers in socio-economically disadvantaged populations. Based on this conclusion – along with the low certainty of findings and the high ROB of the majority of included studies – we suggest that targeted approaches must continue to be pursued, concurrent with efforts to gain a greater understanding of factors associated with their successful implementation and evaluation. This investment is likely to be important to reduce inequalities in health.

## Supplementary Information

**Additional file 1. **

## Data Availability

Data collection templates and data extracted will be made available on reasonable request by contacting the Corresponding Author. Participant data from the included studies is not available.
