# Supplementary material for: Do health education initiatives assist socioeconomically disadvantaged populations? A systematic review and meta-analyses
Source: BMC Public Health. 2023 Mar 8;23:453. doi: 10.1186/s12889-023-15329-z (PMC9996883; doi:10.1186/s12889-023-15329-z)

**Supplementary Appendix**

Supplement to: Karran EL, Grant AR, Moseley GL, et al. Do health education initiatives assist socio-economically disadvantaged populations? A systematic review and meta-analyses

**Contents**

Appendix 1: Search strategies

Appendix 2: Data analysis strategy

Appendix 3: Classification of intervention characteristics

Appendix 4: Risk of bias results

Appendix 5: Publication bias

Appendix 6: Grading of Recommendations Assessment, Development and Evaluation

(GRADE): Summary of Findings for studies in meta-analyses

Appendix 7: Subgroup analyses – Studies with moderate intensity physical activity outcomes

Appendix 8: Subgroup analyses – Studies with cancer screening outcomes

Appendix 9: Meta-analyses – Biomarker Outcomes

Appendix 1: Search strategies

**Search strategy**

1. (promot* or educat* or knowledg* or intervention* or inform* or aware* or communicat*).tw.
2. Exp Health/
3. 1 and 2
4. Vulnerable populations/
5. ((socioeconomic* or socio*economic* or SES or living standard* or standard* of living or economic or educat* or employ* or occupation* or umemploy* or income or wage* or earn* or resourc* OR social condition* or living condition* or population* or patient* or group* or consumer*) adj3 (low or disadvantage* or deprived or underserved or under*served or minorit* or sensitive or insecur* or poor)).mp.
6. Psychosocial Deprivation/
7. (poor or poverty or impover* or unemploy* or non-employ* or non employ*).tw.
8. (depriv* or disadvantag* or needy or underprivileg* or under-privileg* or underserve* or under-serv*).tw.
9. 4 OR 5 OR 6 OR 7 OR 8
10. Randomi$ed controlled trial.pt.
11. controlled clinical trial.pt.
12. randomi$ed.ab.
13. placebo.ab.
14. Clinical Trials as Topic/
15. randomly.ab.
16. trial.ti.
17. (crossover or cross*over).tw.
18. Pragmatic Clinical Trials as Topic/
19. pragmatic clinical trial.pt.
20. 10 or 11 or 12 or 13 or 14 or 15 or 16 or 17 or 18 or 19
21. 3 and 9 and 20
22. Limit: Humans, English Language

**Medline search strategy**

1. (promot* or educat* or knowledg* or inform* or aware* or communicat*).tw.
2. (intervention* or campaign* or initiative* or program* or scheme*).tw.
3. Exp Health/
4. 1 and 2 and 3
5. Vulnerable populations/
6. ((socioeconomic* or socio*economic* or SES or living standard* or standard* of living or economic or educat* or employ* or occupation* or umemploy* or income or wage* or earn* or resource* or social condition* or living condition* or population* or patient* or group* or consumer*) adj3 (low or disadvantage* or deprived or underserved or under*served or minorit* or sensitive or insecur* or poor)).mp.
7. Psychosocial Deprivation/
8. (poor or poverty or impover* or unemploy* or non-employ* or non employ*).tw.
9. (depriv* or disadvantag* or needy or underprivileg* or under-privileg* or underserve* or under-serv*).tw.
10. 5 OR 6 OR 7 OR 8 OR 9
11. Randomized controlled trial/
12. controlled clinical trial/
13. random*.ti,ab.
14. placebo.ab.
15. Clinical Trials as Topic/
16. trial.ti.
17. Cross-over studies/
18. Pragmatic Clinical Trials as Topic/
19. 11 or 12 or 13 or 14 or 15 or 16 or 17 or 18
20. 4 and 10 and 19
21. Limit: Humans, English Language

**Emcare search strategy**

1. (promot* or educat* or knowledg* or inform* or aware* or communicat*).tw.
2. (intervention* or campaign* or initiativ* or program* or scheme*).tw.
3. Exp Health/
4. 1 and 2 and 3
5. Vulnerable populations/
6. ((socioeconomic* or socio*economic* or SES or living standard* or standard* of living or economic or educat* or employ* or occupation* or umemploy* or income or wage* or earn* or resourc* OR social condition* or living condition* or population* or patient* or group* or consumer*) adj3 (low or disadvantage* or deprived or underserved or under*served or minorit* or sensitive or insecur* or poor)).mp.
7. Social isolation/
8. (poor or poverty or impover* or unemploy* or non-employ* or non employ*).tw.
9. (depriv* or disadvantag* or needy or underprivileg* or under-privileg* or underserve* or under-serv*).tw.
10. 5 OR 6 OR 7 OR 8 OR 8 OR 9
11. randomized controlled trial/
12. controlled clinical study/
13. random*.ti,ab.
14. placebo.ab
15. “clinical trial (topic)”/
16. trial.ti
17. crossover procedure/
18. pragmatic trial/
19. 11 or 12 or 13 or 14 or 15 or 16 or 17 or 18
20. 4 and 10 and 19
21. Limit: Humans, English Language

**CENTRAL search strategy**

1. (promot* or educat* or knowledg* or inform* or aware* or communicat*).tw.
2. (intervention* or campaign* or initiativ* or program* or scheme*).tw.
3. Exp Health/
4. 1 and 2 and 3
5. Vulnerable populations/
6. ((socioeconomic* or socio*economic* or SES or living standard* or standard* of living or economic or educat* or employ* or occupation* or umemploy* or income or wage* or earn* or resourc* OR social condition* or living condition* or population* or patient* or group* or consumer*) adj3 (low or disadvantage* or deprived or underserved or under*served or minorit* or sensitive or insecur* or poor)).mp.
7. Psychosocial Deprivation/
8. (poor or poverty or impover* or unemploy* or non-employ* or non employ*).tw.
9. (depriv* or disadvantag* or needy or underprivileg* or under-privileg* or underserve* or under-serv*).tw.
10. 5 OR 6 OR 7 OR 8 OR 9
11. Randomized controlled trial/
12. controlled clinical trial/
13. random*.ti,ab.
14. placebo.ab.
15. Clinical Trials as Topic/
16. trial.ti.
17. Cross-over studies/
18. Pragmatic Clinical Trials as Topic/
19. 11 or 12 or 13 or 14 or 15 or 16 or 17 or 18
20. 4 and 10 and 19

Appendix 2: Data analysis strategy

*Stage 1: Meta-analyses*

We examined the included studies for clinical and methodological heterogeneity so as to identify data suitable for pooling in meta-analyses. We sub-grouped the studies according to the health focus of the intervention and considered factors such as study design, outcome measures, follow-up duration, and comparison groups. Meta-analysis was determined to be appropriate when there were three or more similar studies with homogenous outcomes. We used Comprehensive Meta-Analysis (version 3)(129) software to conduct meta-analyses for studies we considered to be sufficiently homogenous, using random effects models. Available data for study observations (e.g. means, standard deviations, percentages) or effect estimates (e.g. standardised mean difference, odds ratios) were entered into the meta-analysis software and pooled estimates of effect were reported in terms of standardised effect size measure Hedges g. We assessed statistical heterogeneity between the studies in the meta-analyses using I^2^ and Tau^2^ statistics. We applied a cut-off of I^2^ ≤ 75% to determine ‘acceptable’ heterogeneity, as recommended by Higgins et al. (2019) and report Tau^2^ to describe the distribution of the true effect.(130, 131) Subgroup analyses (as pre-specified in the protocol) were carried out where possible.

We evaluated the quality of the evidence of the included studies and rated the strength of recommendations using the Grading of Recommendations Assessment, Development and Evaluation (GRADE) framework.(22) The quality of evidence was downgraded according to the following criteria: risk of bias, inconsistency, indirectness, imprecision and publication bias. Publication bias was assessed by visual inspection of a funnel plot; Egger’s test was applied if there were 10 or more studies in the meta-analysis.(23)

*Stage 2: Vote-counting*

We summarised the overall effectiveness of interventions for our primary and secondary outcomes using a vote-counting approach. When studies specified a single primary outcome, the intervention effect was determined by that outcome. When studies had two or more outcomes, a decision rule was applied to identify a single outcome from which to classify intervention benefit:

DECISION RULE FOR OUTCOME SELECTION:

1. Where the study specifies a single primary outcome – use this outcome

2. Where there is more than one primary outcome:

(i) Use the most outcome that is most relevant to the focus of the intervention (i.e. consider the content validity of the outcome measurement)

(ii) If it is uncertain which outcome is most relevant or they seem equally relevant, randomly select a single outcome (from the primary outcomes).

3. If there is more than one outcome and primary/secondary outcomes are not specified:

(i) Use the most outcome that is most relevant to the focus of the intervention.

(ii) If it is unclear which outcome is most relevant or they seem equally relevant, randomly select a single outcome (from all outcomes)

4. Do this for studies with behavioural outcomes and all studies with biomarker outcomes

Two authors will apply this decision rule to all studies and independently vote for which outcome to use. Voters will be given summary details of the participant group and focus of the intervention but will be blinded to the results for each outcome. Votes will be recorded on an excel spreadsheet and will be compared. If there is discrepancy, a 3rd author (EK) will vote to decide on which outcome to use.

Studies were then classified using a standardised binary metric assigned according to on the basis of statistical the observed direction of effect. The number of effects showing benefit, as a proportion of the total number of studies was then calculated (without consideration of statistical significance nor the size of the effect), and a confidence interval was determined using the Agresti-Coull interval method recommended for large sample sizes

**Appendix 3:** Classification of intervention characteristics

| **Classification** | **Definition** |
| --- | --- |
| **Setting** | ‘Community based’; ‘home-based’; conducted in a ‘health clinic’ or ‘community centre’ |
| **Type** | ‘Individual’ sessions’; ‘group’ sessions or ‘both’; provision of ‘resources only’ or ‘community-level’ education |
| **Dose** | Cumulative intervention dose was determined by duration, frequency (number of contacts) and amount (number of hours)(25)  ‘Low’ dose: ≤2 months duration, ≤2 contacts, and ≤6 hours  ‘Moderate’ dose: <4 months duration, 3-10 contacts and <10 hours  ‘High’ dose: >3 months duration, ≥10 contacts, ≥ 10 hours  If any of the dosage sub-categories were exceeded, the intervention was classified as the higher dosage level |
| **Complexity** | ‘Education only’; ‘education plus peer support’ or ‘complex’.  *Complex* interventions involved the standardised delivery of an intervention component that was not educational. E.g. smoking cessation intervention included provision of nicotine patches; or weight loss education that also included exercise classes |
| **Follow-up** | ‘Short term’: </=3 months; ‘medium term’: 3-6 months; ‘long term’: >6 months |
| **Outcomes** | ‘Self-report’; ‘objective’ or ‘both’ (relevant to behavioural outcomes only) |

Appendix 4: Risk of bias results

| **Author, year** | **Selection bias** | | **Performance Bias** | **Detection bias** | | **Attrition bias** | | **Reporting bias** | **Other bias** | | **OVERALL BIAS** |
| --- | --- | --- | --- | --- | --- | --- | --- | --- | --- | --- | --- |
|  | **Random sequence generation** | **Allocation concealment** | **Blinding of participants** | **Blinding of outcome assessment (subjective outcomes)** | **Blinding of outcome assessment (objective outcomes)** | **Appropriate method used for missing data** | **Number lost to follow up** | **Selective reporting of outcomes** | **Free of relevant baseline imbalances** | **Intent to treat analysis** |  |
| Abiyu (2020)(58) |  |  |  |  | - |  |  |  |  |  |  |
| Acharya (2015)(59) |  |  |  |  | - |  |  |  |  |  |  |
| Alegria (2014)(61) |  |  |  |  | - |  |  |  |  |  |  |
| Alias (2021)(62) |  |  |  |  | - |  |  |  |  |  |  |
| Almabadi (2021)(60) |  |  |  |  | - |  |  |  |  |  |  |
| Alvarenga (2020)(63) |  |  |  | **-** |  | **-** | **-** |  |  | **-** |  |
| Andrews (2016)(64) |  |  |  |  |  |  |  |  |  |  |  |
| Annan (2017)(65) |  |  |  |  | **-** |  |  |  |  |  |  |
| Avila (1994)(37) |  |  |  |  |  |  |  |  |  |  |  |
| Bagner (2016)(66) |  |  |  | **-** |  |  |  |  |  |  |  |
| Baranowski (1990)(67) |  |  |  |  |  |  |  |  |  |  |  |
| Barry (2022)(68) |  |  |  |  |  |  |  | **-** |  | **-** |  |
| Befort (2016)(69) |  |  |  | **-** |  |  |  |  |  |  |  |
| Berman (1995)(70) |  |  |  |  | **-** |  |  |  |  |  |  |
| Bray (2013)(71) |  |  |  | **-** |  |  |  |  |  |  |  |
| Brooking (2012)(53) |  |  |  |  | **-** |  |  |  |  |  |  |
| Brooks (2018)(72) |  |  |  |  |  |  |  |  |  |  |  |
| Brown (2013)(73) |  |  |  | **-** |  |  |  |  |  |  |  |
| Byrd (2013)(32) |  |  |  |  |  |  |  |  |  |  |  |
| Cahill (2018)(74) |  |  |  | **-** |  |  |  |  |  |  |  |
| Calderon-Mora (2020)(44) |  |  |  |  | **-** |  |  |  |  |  |  |
| Childs (1997)(75) |  |  |  | **-** |  |  |  |  |  |  |  |
| Cibulka (2011)(76) |  |  |  |  | **-** |  |  |  |  |  |  |
| Curry (2003)(77) |  |  |  |  | **-** |  |  |  |  |  |  |
| Damush (2003)(78) |  |  |  |  | **-** |  |  |  |  |  |  |
| Dawson-McClure (2014)(79) |  |  |  |  | **-** |  |  |  |  |  |  |
| Dela Cruz (2012)(80) |  |  |  | **-** |  |  |  |  |  |  |  |
| Doorenbos (2011)(43) |  |  |  | **-** |  |  |  |  |  |  |  |
| El-Mohandes (2003)(81) |  |  |  |  |  |  |  |  |  |  |  |
| El-Mohandes (2010)(82) |  |  |  |  |  |  |  |  |  |  |  |
| Emmons (2001)(83) |  |  |  | **-** |  |  |  |  |  |  |  |
| Falbe (2015)(84) |  |  |  | **-** |  |  |  |  |  |  |  |
| Fernandez-Jimenez (2020)(85) |  |  |  |  |  |  |  |  |  |  |  |
| Fiks (2017)(86) |  |  |  |  | **-** |  |  |  |  |  |  |
| Fitzgibbon (2004)(41) |  |  |  |  | **-** |  |  |  |  |  |  |
| Fitzgibbon (1996)(87) |  |  |  |  | **-** |  |  |  |  |  |  |
| Fox (1999)(88) |  |  |  |  | **-** |  |  |  |  |  |  |
| Gathirua-Mwangi (2016)(33) |  |  |  |  |  |  |  |  |  |  |  |
| Gielen (1997)(89) |  |  |  |  |  |  |  |  |  |  |  |
| Hayashi (2010)(40) |  |  |  |  |  |  |  |  |  |  |  |
| Hesselink (2012)(90) |  |  |  |  |  |  |  |  |  |  |  |
| Hillemeier (2008)(39) |  |  |  |  |  |  |  |  |  |  |  |
| Hoodbhoy (2021) |  |  |  |  |  |  |  |  |  | **-** |  |
| Hooper (2017)(91) |  |  |  | **-** |  |  |  |  |  |  |  |
| Hovell (2008)(29) |  |  |  |  |  |  |  |  |  |  |  |
| Hunt (1976)(92) |  |  |  | **-** |  |  |  |  |  |  |  |
| Jacobson (1999)(93) |  |  |  | **-** |  |  |  |  |  |  |  |
| Janicke (2008)(94) |  |  |  | **-** |  |  |  |  |  |  |  |
| Kalichman (2000)(42) |  |  |  |  | **-** |  |  |  |  |  |  |
| Kasari (2014)(96) |  |  |  | **-** |  |  |  |  |  |  |  |
| Katz (2007)(34) |  |  |  |  | **-** |  |  |  |  |  |  |
| Kelly (1994)(97) |  |  |  |  | **-** |  |  |  |  |  |  |
| Keyserling (2008)(30) |  |  |  | **-** |  |  |  |  |  |  |  |
| Khare (2012)(27) |  |  |  |  |  |  |  |  |  |  |  |
| Khare (2014)(28) |  |  |  |  |  |  |  |  |  |  |  |
| Kim (2014)(54) |  |  |  | **-** |  |  |  |  |  |  |  |
| King (2013)(38) |  |  |  |  | **-** |  |  |  |  |  |  |
| Kisioglu (2004)(55) |  |  |  | **-** |  |  |  |  |  |  |  |
| Kreuter (2005)(35) |  |  |  |  | **-** |  |  |  |  |  |  |
| Kreuter (2010)(45) |  |  |  |  | **-** |  |  |  |  |  |  |
| Krieger (2005)(99) |  |  |  |  | **-** |  |  |  |  |  |  |
| Kulathinal (2019)(100) |  |  |  |  | **-** |  | **-** |  | **-** |  |  |
| Lutenbacher (2018)(101) |  |  |  |  | **-** |  |  |  |  |  |  |
| Maldonaldo (2020)(102) | **-** | **-** |  |  | **-** |  |  |  |  |  |  |
| Manandhar (2004)(103) |  |  |  |  | **-** |  |  |  |  |  |  |
| Martin (2011)(104) |  |  |  | **-** |  |  |  |  |  |  |  |
| McConnell (2016)(106) |  |  |  |  | **-** |  |  |  |  |  |  |
| McGilloway (2014)(107) |  |  |  |  |  |  |  |  |  |  |  |
| Miller (2013)(108) |  |  |  | **-** |  |  |  |  |  |  |  |
| Murthy (2019)(109) |  |  |  |  | **-** |  |  |  |  |  |  |
| Pandey (2007)(110) |  |  |  |  | **-** |  |  |  |  |  |  |
| Parra-Medina (2011)(31) |  |  |  |  | **-** |  |  |  |  |  |  |
| Phillips (2014)(111) |  |  |  | **-** |  |  |  |  |  |  |  |
| Pitchik (2021)(112) |  |  |  |  | **-** |  |  |  |  |  |  |
| Polomoff (2022)(113) |  |  |  |  | **-** |  |  |  |  |  |  |
| Reijneveld (2003)(114) |  |  |  |  | **-** |  |  |  |  |  |  |
| Reisine (2012)(115) |  |  |  |  | **-** |  |  |  |  |  |  |
| Ridgeway (2022)(116) |  |  |  |  | **-** |  |  |  |  |  |  |
| Robinson (2002)(117) |  |  |  |  | **-** |  |  |  |  |  |  |
| Ryser (2004)(118) |  |  |  |  | **-** |  |  |  |  |  |  |
| Saleh (2018)(119) |  |  |  | **-** |  |  |  |  |  |  |  |
| Santa Maria (2021)(120) |  |  |  |  |  | **-** |  |  |  |  |  |
| Segal-Isaacson (2006)(121) |  |  |  |  | **-** |  |  |  |  |  |  |
| Seguin Fowler (2020)(122) | **-** | **-** |  |  |  |  |  |  |  |  |  |
| Simmons (2022)(123) |  |  |  |  | **-** |  |  |  |  |  |  |
| Smith (2021)(124) |  |  |  |  | **-** |  |  |  |  |  |  |
| Staten (2004)(56) |  |  |  |  |  |  |  |  |  |  |  |
| Steptoe (2003)(125) |  |  |  | **-** |  |  |  |  |  |  |  |
| Suhadi (2018)(57) |  |  |  | **-** |  |  |  |  |  |  |  |
| Valdez (2016)(36) |  |  |  |  | **-** |  |  |  |  |  |  |
| Wiggins (2005)(126) |  |  |  |  | **-** |  |  |  |  |  |  |
| Xu (2019)(127) |  |  |  | **-** |  |  |  |  |  |  |  |
| Zoellner (2016)(26) |  |  |  |  |  |  |  |  |  |  |  |

|  | Low risk of bias |  | Unclear risk of bias |  | High risk of bias | - | Not applicable |
| --- | --- | --- | --- | --- | --- | --- | --- |

Appendix 5: Publication bias

**Figure (a). Funnel plot of standard error by Hedges’ g for trials included in the meta-analysis of studies with *moderate intensity physical activity* outcomes. (26-31) The open circles represent observed studies and the open diamond represents the observed effect size.**


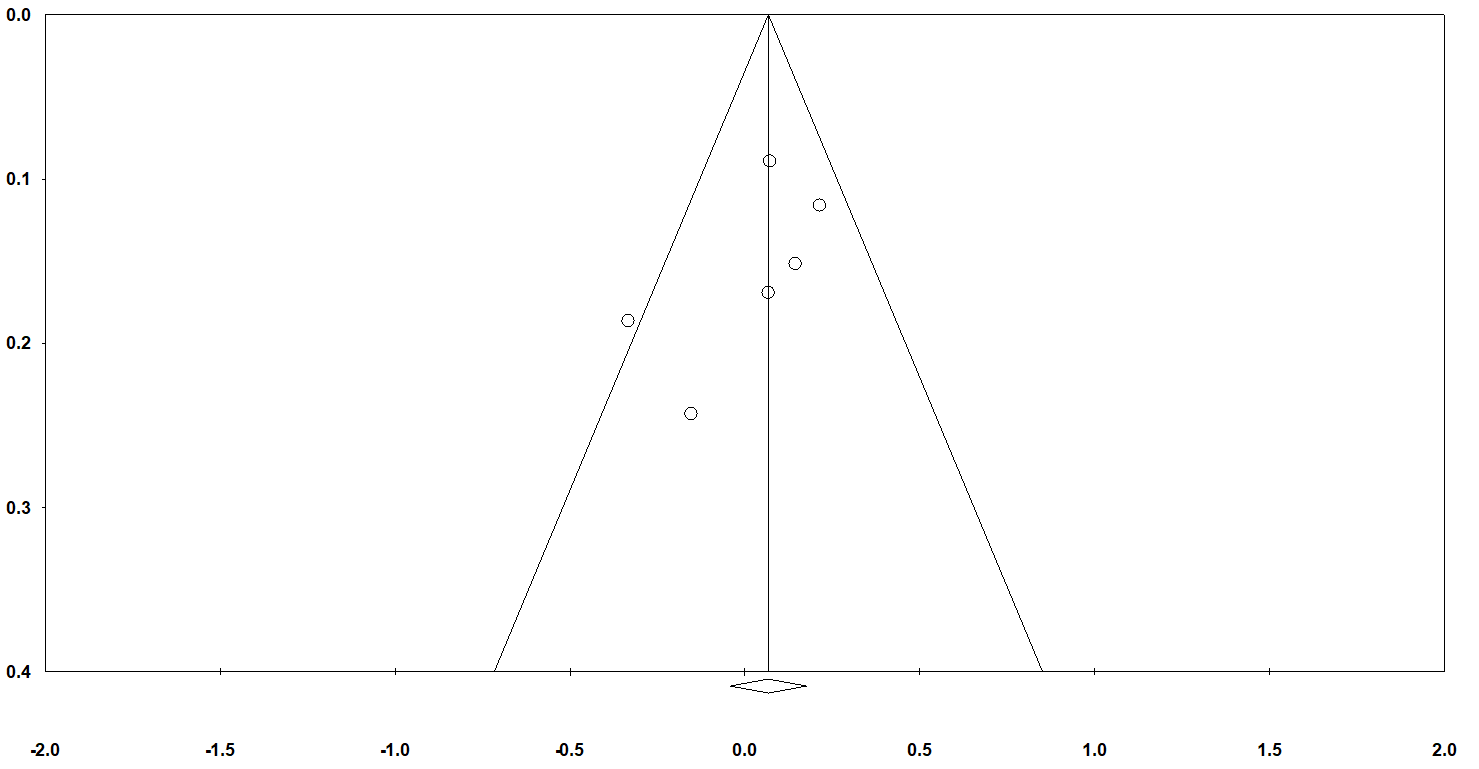


Standard error

Hedges g

**Figure (b). Funnel plot of standard error by Hedges’ g for trials included in the meta-analysis of studies with *cancer screening* outcomes.(32-36) The open circles represent observed studies and the open diamond represents the observed effect size.**


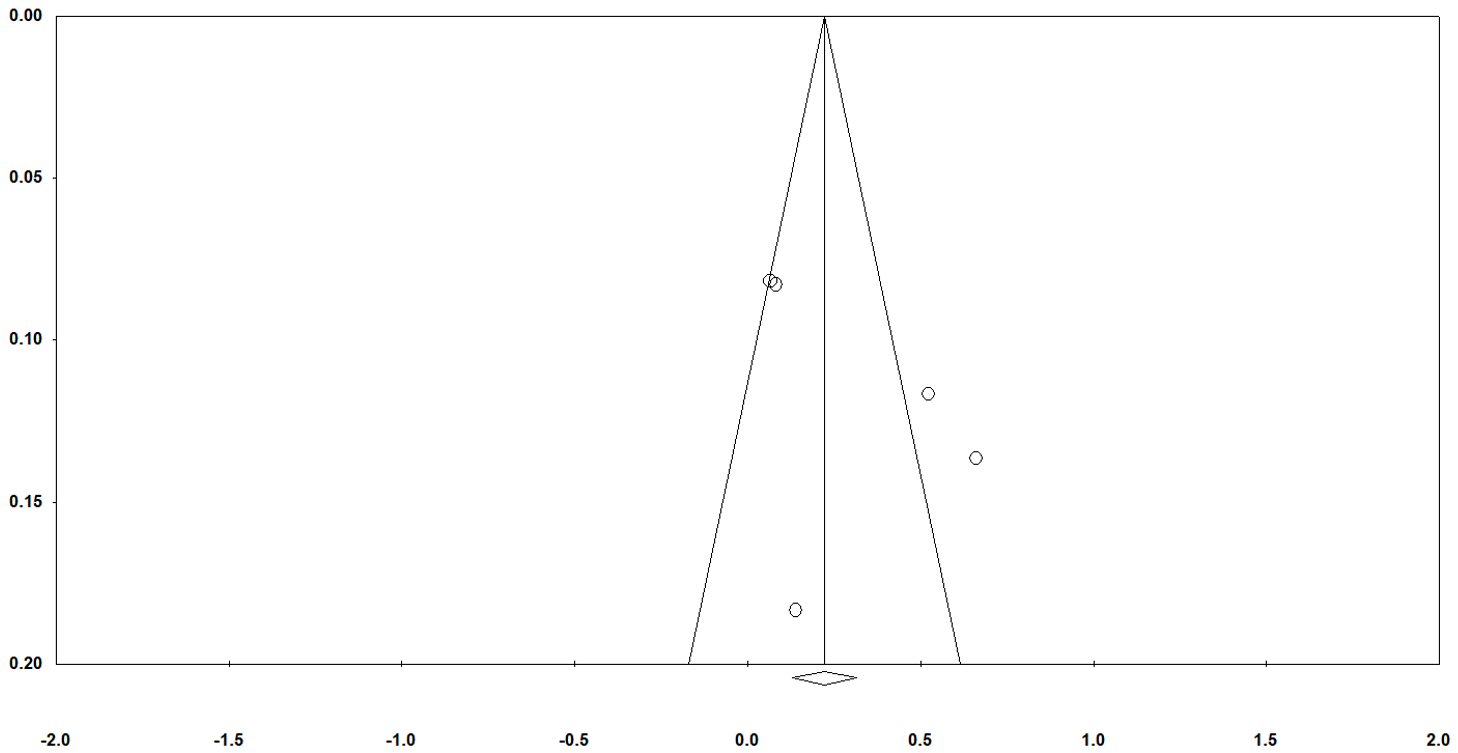


Standard error

Hedges g

**Figure (c). Funnel plot of standard error by Hedges’ g for trials included in the meta-analysis of studies with *body mass index* outcomes.(28, 53, 55-57)** **The open circles represent observed studies and the open diamond represents the observed effect size.**


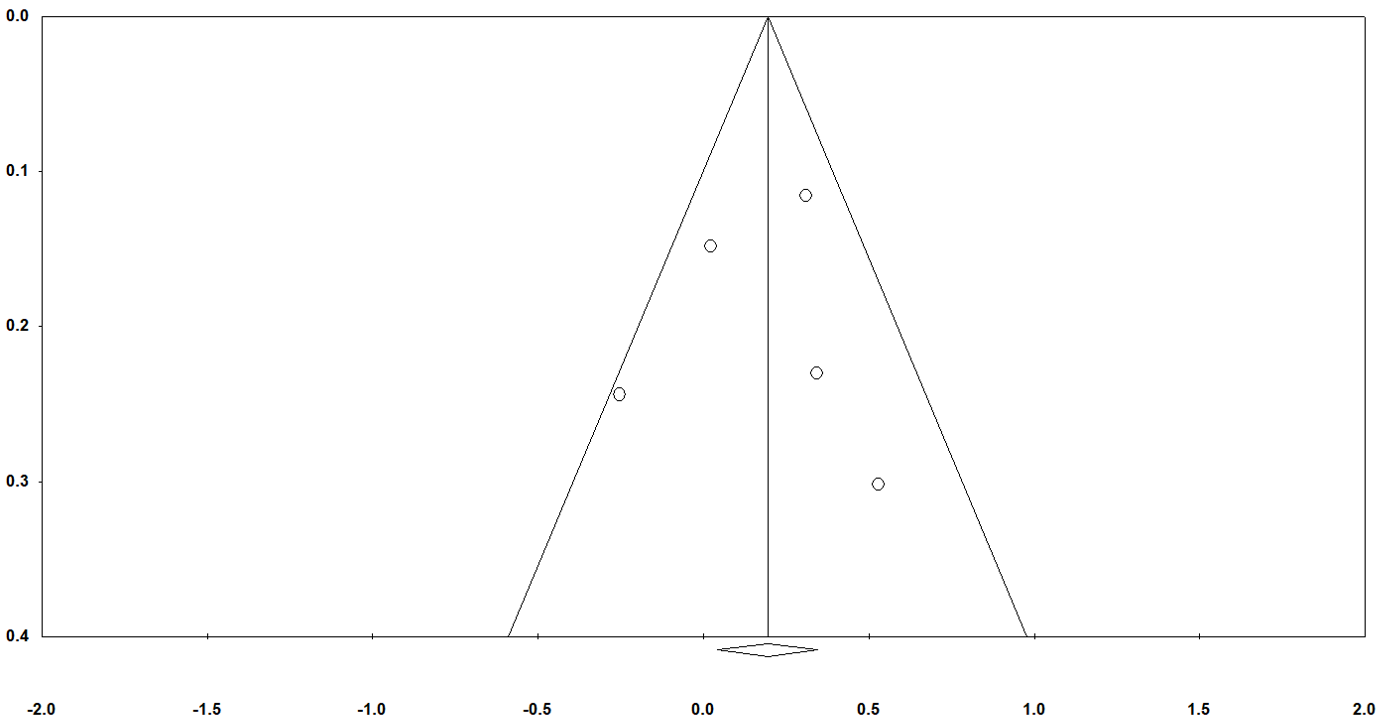


Standard error

Hedges g

**Figure (d). Funnel plot of standard error by Hedges’ g for trials included in the meta-analysis of studies with *blood pressure* outcomes. (30, 53-55, 57)**  **The open circles represent observed studies and the open diamond represents the observed effect size.**


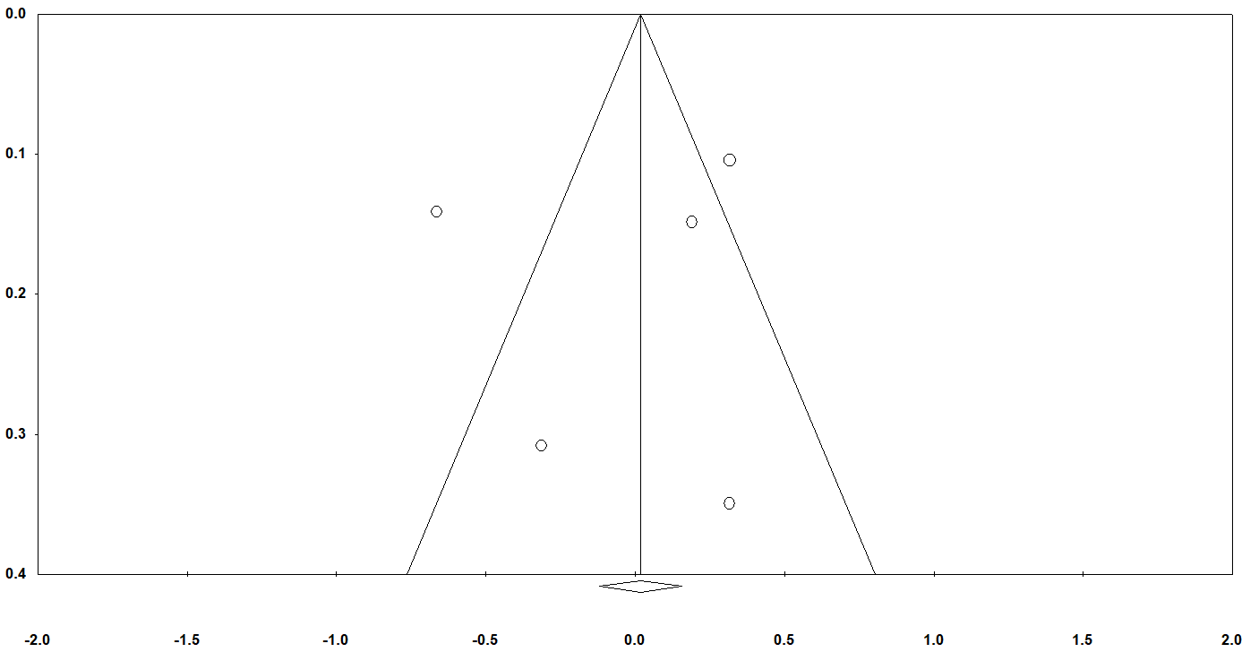


Standard error

Hedges g

**Figure (e). Funnel plot of standard error by Hedges’ g for trials included in the meta-analysis of studies with *cholesterol* outcomes.** **(28, 30, 53, 57) The open circles represent observed studies and the open diamond represents the observed effect size.**


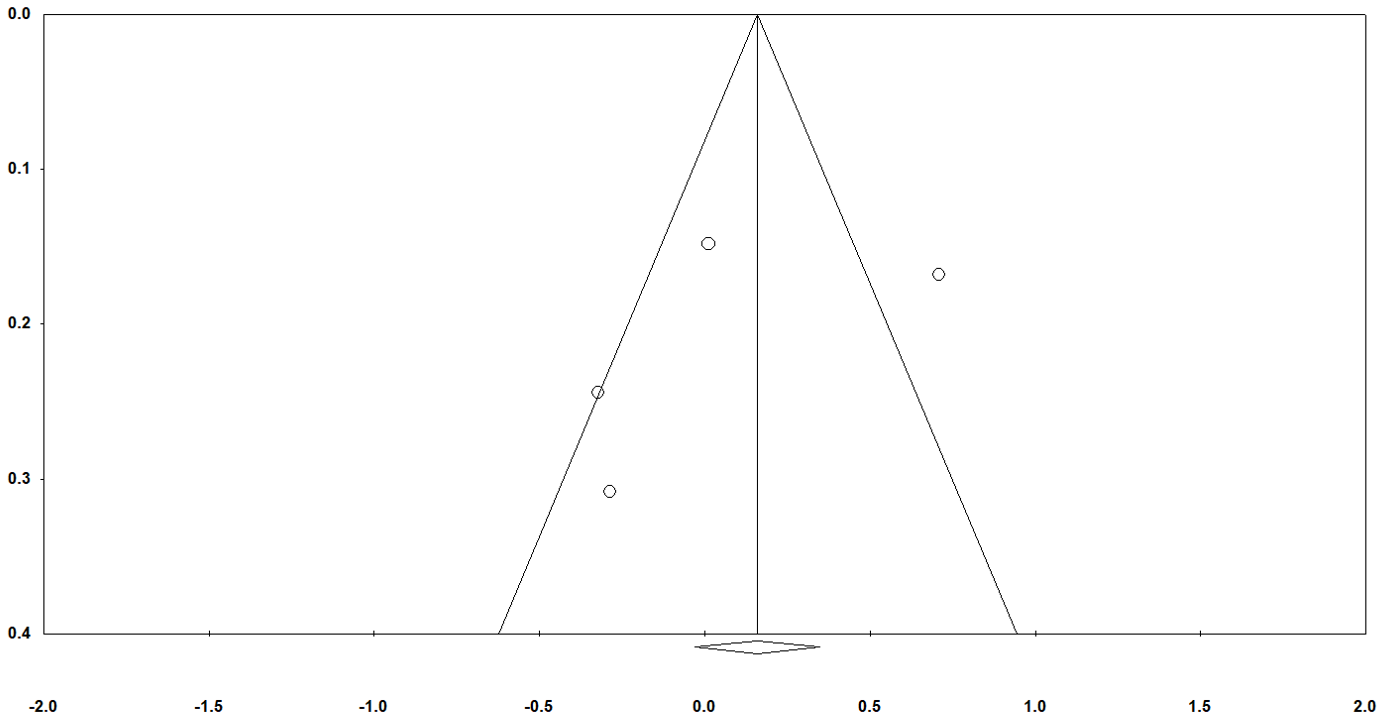


Standard error

Hedges g

**Figure (f). Funnel plot of standard error by Hedges’ g for trials included in the meta-analysis of studies with *blood glucose* outcomes. (28, 53, 56, 57)**  **The open circles represent observed studies and the open diamond represents the observed effect size.**


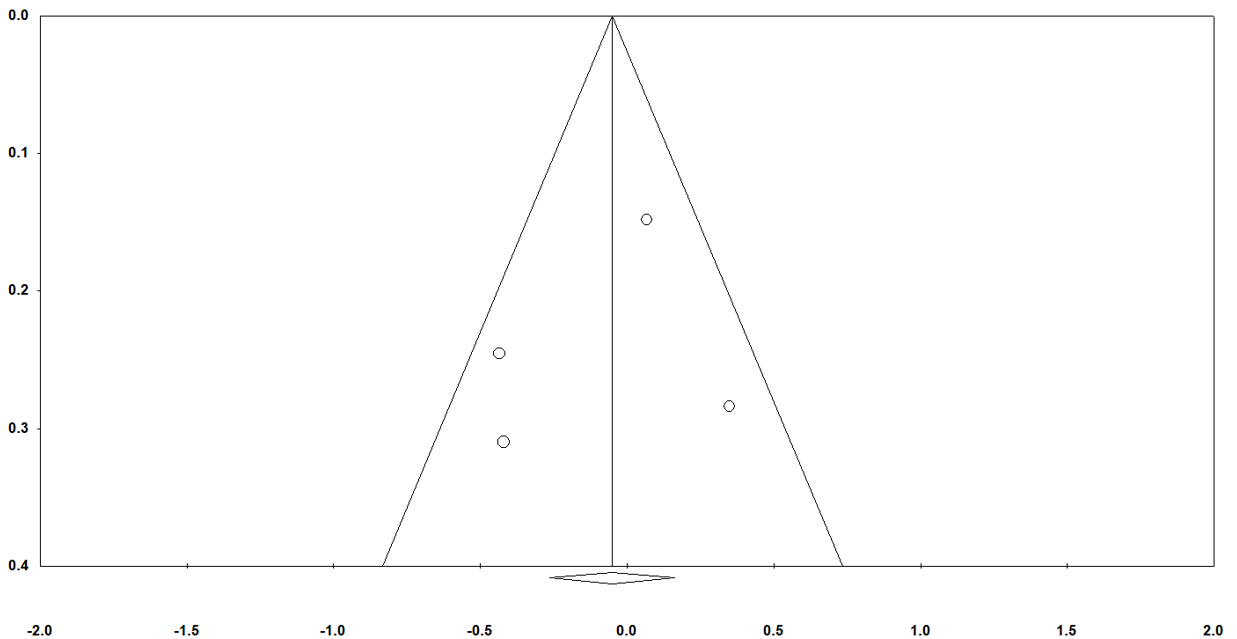


Standard error

Hedges g

Appendix 6: Grading of Recommendations Assessment, Development and Evaluation (GRADE): Summary of Findings for studies included in meta-analyses

|  | **Certainty Assessment** | | | | | | | **No of participants** | **Effect size**  **Hedges (95%CI)** | **Certainty** |
| --- | --- | --- | --- | --- | --- | --- | --- | --- | --- | --- |
| **No of studies** | **Study design** | **Risk of bias** | | **Inconsistency** | **Indirectness** | **Imprecision** | **Publication bias** |  |  |  |
| **Outcome: Moderate intensity physical activity** (29-34) | | | | | | | | | | |
| 6 studies | +4 | -1 | | -0 | -0 | -0 | -0 |  |  | 3 |
| Hovell 2008 | RCT | High | Serious risk of bias | I^2^ 31%  Inconsistency does not appear to be an issue | Does not appear to be an issue for any of the studies | No serious concern | Undetected | 1330 | 0.05 (-0.09-0.19) | Moderate certainty |
| Keyserling 2008 | RCT | High |  |  |  |  |  |  |  |  |
| Khare 2012 | RCT | High |  |  |  |  |  |  |  |  |
| Khare 2014 | RCT | High |  |  |  |  |  |  |  |  |
| Parra-Medina 2011 | RCT | High |  |  |  |  |  |  |  |  |
| Zoellner 2016 | RCT | Unclear |  |  |  |  |  |  |  |  |
| **Outcome: Cancer screening** (35-39) | | | | | | | | | | |
| 5 studies | +4 | -1 | | -2 | -0 | -1 | -0 |  |  | 0 |
| Byrd 2013 | RCT | Unclear | Serious risk of bias | I^2^ 83%  Severe inconsistency is present | Does not appear to be an issue | * | Undetected | 2388 | 0.29 (0.05-0.52) | Very low certainty |
| Gathirua-Mwangi 2016 | RCT | Unclear |  |  |  |  |  |  |  |  |
| Katz 2007 | RCT | High |  |  |  |  |  |  |  |  |
| Kreuter 2005 | RCT | High |  |  |  |  |  |  |  |  |
| Valdez 2016 | RCT | High |  |  |  |  |  |  |  |  |
| **Outcome: Body mass index** (31, 40-43) | | | | | | | | | | |
| 5 studies | +4 | -1 | | -1 | -0 | -0 | -0 |  |  | 2 |
| Brooking 2012 | RCT | High | Serious risk of bias | I^2^ 46%  Some inconsistency exists | Does not appear to be an issue | No serious concern | Undetected | 832 | 0.18 (-0.05-0.41) | Low certainty |
| Khare 2014 | RCT | High |  |  |  |  |  |  |  |  |
| Kisioglu 2004 | RCT | High |  |  |  |  |  |  |  |  |
| Staten 2004 | RCT | High |  |  |  |  |  |  |  |  |
| Suhadi 2018 | RCT (Cluster) | High |  |  |  |  |  |  |  |  |
| **Outcome: Blood pressure** (33, 40, 41, 43, 44) | | | | | | | | | | |
| 5 studies | +4 | -1 | | -2 | -0 | -0 | -0 |  |  | 1 |
| Brooking 2012 | RCT | High | Serious risk of bias | I^2^ 89%  Severe inconsistency is present | Does not appear to be an issue | No serious concern | Undetected | 1202 | -0.04 (-0.49-0.41) | Very low certainty |
| Kim 2014 | RCT | High |  |  |  |  |  |  |  |  |
| Kisioglu 2004 | RCT | High |  |  |  |  |  |  |  |  |
| Keyserling 2008 | RCT | High |  |  |  |  |  |  |  |  |
| Suhadi 2018 | RCT | High |  |  |  |  |  |  |  |  |
| **Outcome: Cholesterol** (31, 33, 40, 43) | | | | | | | | | | |
| 4 studies | +4 | -1 | | -2 | -0 | -1 | -0 |  |  | 0 |
| Brooking 2012 | RCT | High | Serious risk of bias | I^2^ 83%  Severe inconsistency is present | Does not appear to be an issue | * | Undetected | 439 | 0.06 (-0.43-0.55) | Very low certainty |
| Keyserling 2008 | RCT | High |  |  |  |  |  |  |  |  |
| Khare 2014 | RCT | High |  |  |  |  |  |  |  |  |
| Suhadi 2018 | RCT | High |  |  |  |  |  |  |  |  |
| **Outcome: Blood glucose** (31, 40, 42, 43) | | | | | | | | | | |
| 4 studies | +4 | -1 | | -1 | -0 | -0 | -0 |  |  | 2 |
| Brooking 2012 | RCT | High | Serious risk of bias | I^2^ 54%  Some inconsistency exists | Does not appear to be an issue | No serious concern | Undetected | 412 | -0.09 (-0.43-0.25) | Low certainty |
| Khare 2014 | RCT | High |  |  |  |  |  |  |  |  |
| Staten 2004 | RCT | High |  |  |  |  |  |  |  |  |
| Suhadi 2018 | RCT | High |  |  |  |  |  |  |  |  |

Appendix 7: Subgroup analyses – Studies with moderate intensity physical activity outcomes

Subgroup analysis of studies with complex or ‘non-complex’ interventions were possible. Four studies (n=1006 participants) implemented ‘complex’ interventions(26-29). The pooled effect was 0.10 (95% CI = -0.03-0.22; Tau^2^ = 0.00) (figure a). The I^2^ value of 0% indicates homogeneity across trial results. Two studies (n=324 participants) implemented ‘non-complex’ interventions – either education-only(31) or ‘education plus peer support’.(30) The pooled effect of these studies was -0.08 (95% CI = -0.55-0.39; Tau^2^ = 0.09) (figure b). The I^2^ value of 74% indicates a substantial degree of heterogeneity across study results.

Figure a). Complex interventions (n=4)


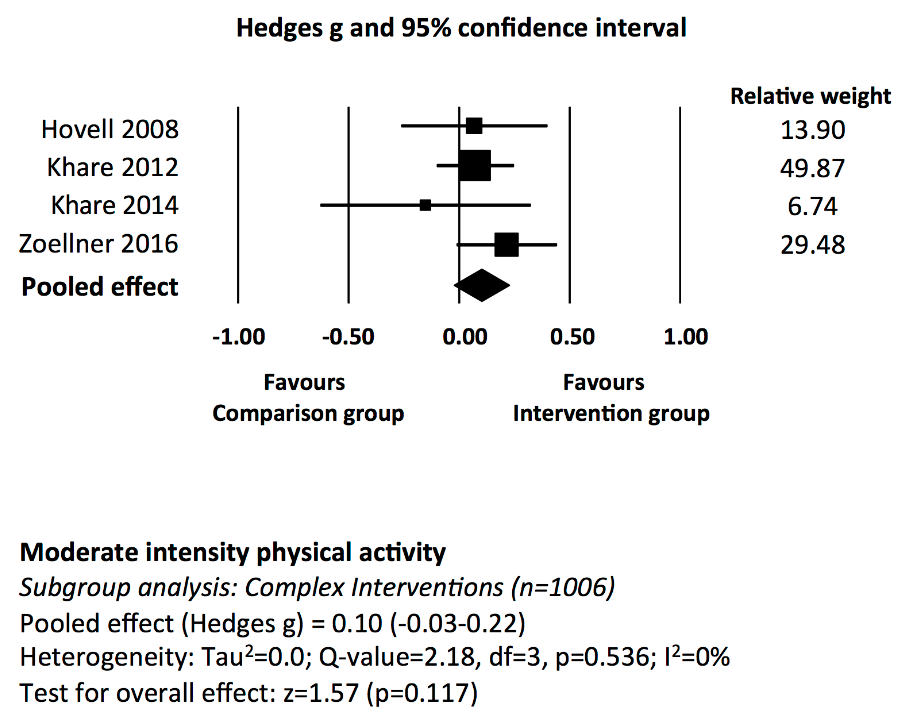


Figure b). ‘Non-complex’ interventions (n=2)


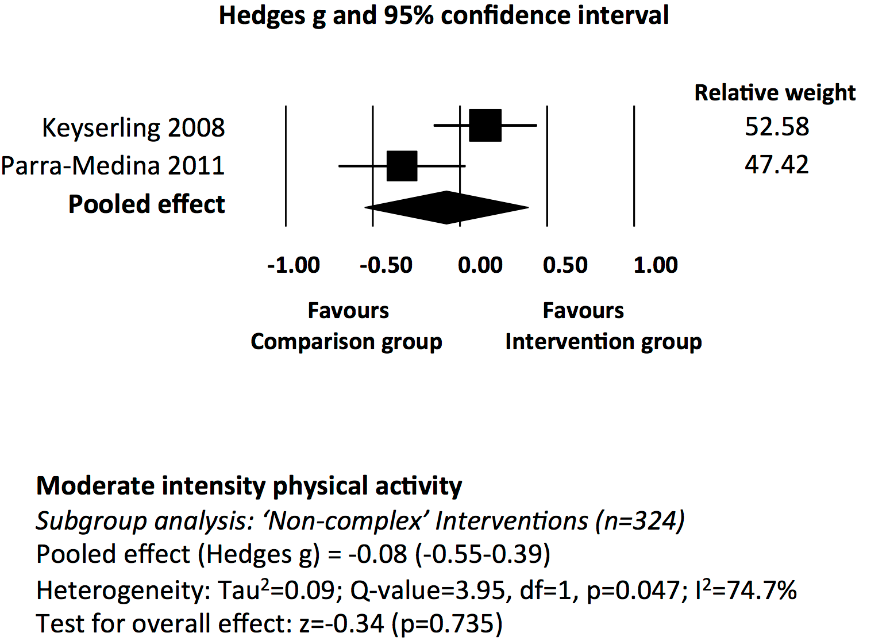


Appendix 8: Subgroup analyses – Studies with cancer screening outcomes

Subgroup analysis of studies with moderate or low-dose interventions were possible. Two studies (n= 1203 participants) delivered interventions that were classified as ‘moderate dose’.(34, 35) The pooled effect was 0.30 (95% CI = -0.14-0.73; Tau^2^ = 0.09) (figure a). The I^2^ value of 89% indicates a considerable degree of heterogeneity across trial results. Three studies (n=1185 participants) delivered interventions that were classified as ‘low dose’.(32, 33, 36) The pooled effect was 0.29 (95% CI = -0.10-0.68; Tau^2^ = 0.10 (figure b). The I^2^ value of 86% indicates a considerable degree of heterogeneity across trial results.

**Figure a**. Low dose interventions (n=3)


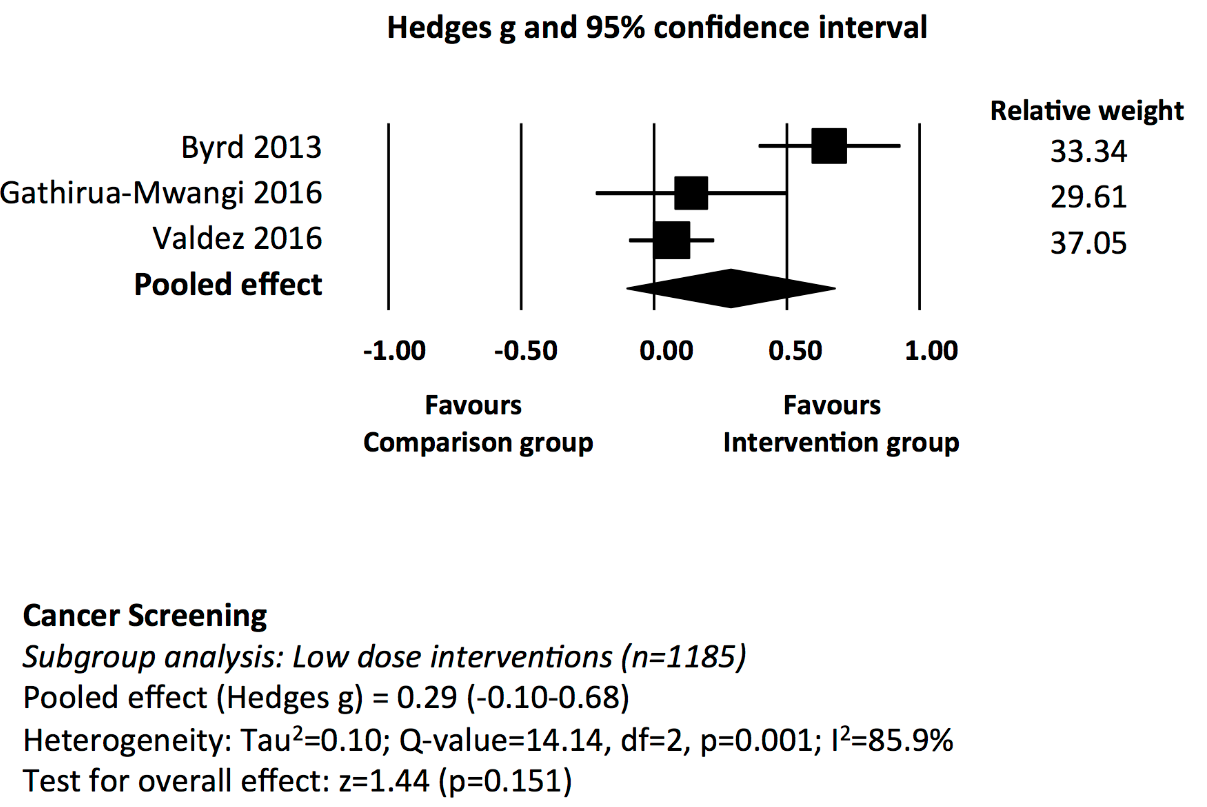


**Figure b.** Moderate dose interventions (n=2)


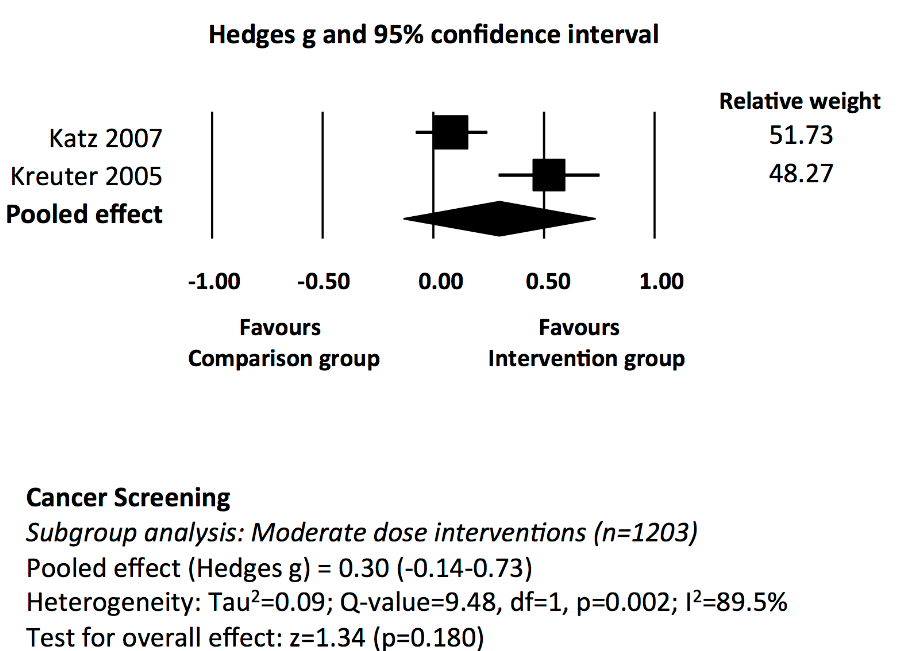


Appendix 9: Meta-analyses – Biomarker Outcomes

Ten studies had body mass index outcomes; eight studies had blood pressure outcomes; eight studies had blood glucose outcomes; and eleven studies had cholesterol outcomes. We did not undertake a meta-analysis of the three studies with blood insulin outcomes since these studies were also included in the larger meta-analysis of blood glucose outcomes.

**Body Mass Index**

Five of ten studies were considered to have adequate methodological and clinical heterogeneity to pool results (n=832).(28, 53, 55-57) We down-graded certainty in the evidence by two levels due to risk of bias and inconsistency. There is low certainty that the pooled effect of educational interventions, when compared to minimal/no intervention or a control intervention, is 0.18 [95% CI = -0.05-0.41; Tau^2^ = 0.03] (figure a). The I^2^ value of 46% indicates moderate heterogeneity across trial results.

**Figure a.** The effectiveness of educational interventions at improving body mass index in socio-economically disadvantaged populations: random effects meta-analysis


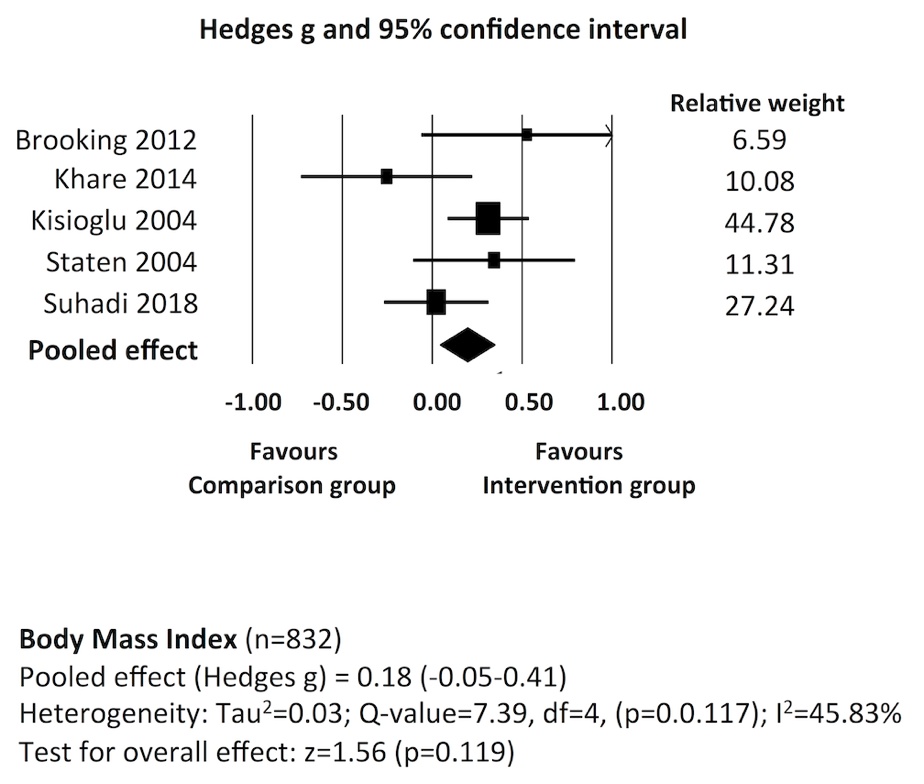


**Blood Pressure**

Five of eight studies were considered to have adequate methodological and clinical heterogeneity to pool results (n=1202).(30, 53-55, 57) We down-graded certainty in the evidence by three levels due to risk of bias and inconsistency (two levels). There is very low certainty that the pooled effect of educational interventions, when compared to minimal/no intervention, is -0.04 (95% CI = -0.49-0.41; Tau^2^ = 0.22) (figure b). The I^2^ value of 89% indicates a considerable degree of heterogeneity across trial results.

**Figure b**. The effectiveness of educational interventions at improving blood pressure in socio-economically disadvantaged populations: random effects meta-analysis


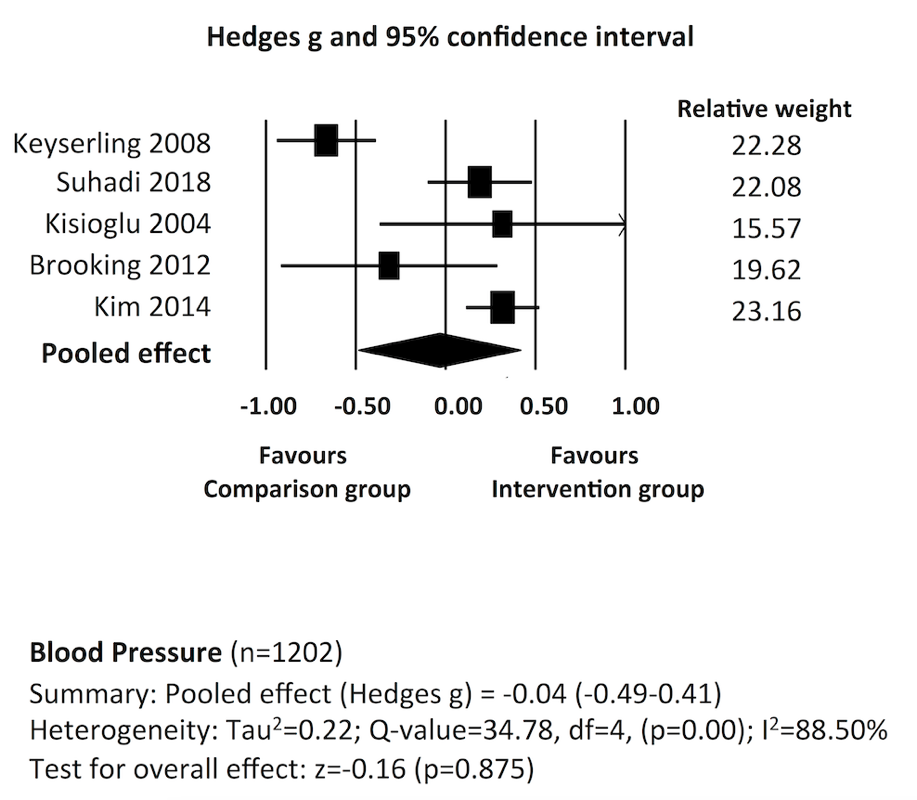


**Blood Glucose**

Four of eight studies were considered to have adequate methodological and clinical heterogeneity to pool results (n=412).(28, 53, 56, 57) We down-graded certainty in the evidence by two levels due to risk of bias and inconsistency. There is low certainty that the pooled effect of educational interventions, when compared to minimal/no intervention or a control intervention, is -0.09 (95% CI = -0.43-0.25; Tau^2^ = 0.06) (figure c). The I^2^ value of 54% indicates a moderate degree of heterogeneity across study results.

**Figure c.** The effectiveness of educational interventions at improving blood glucose in socio-economically disadvantaged populations: random effects meta-analysis


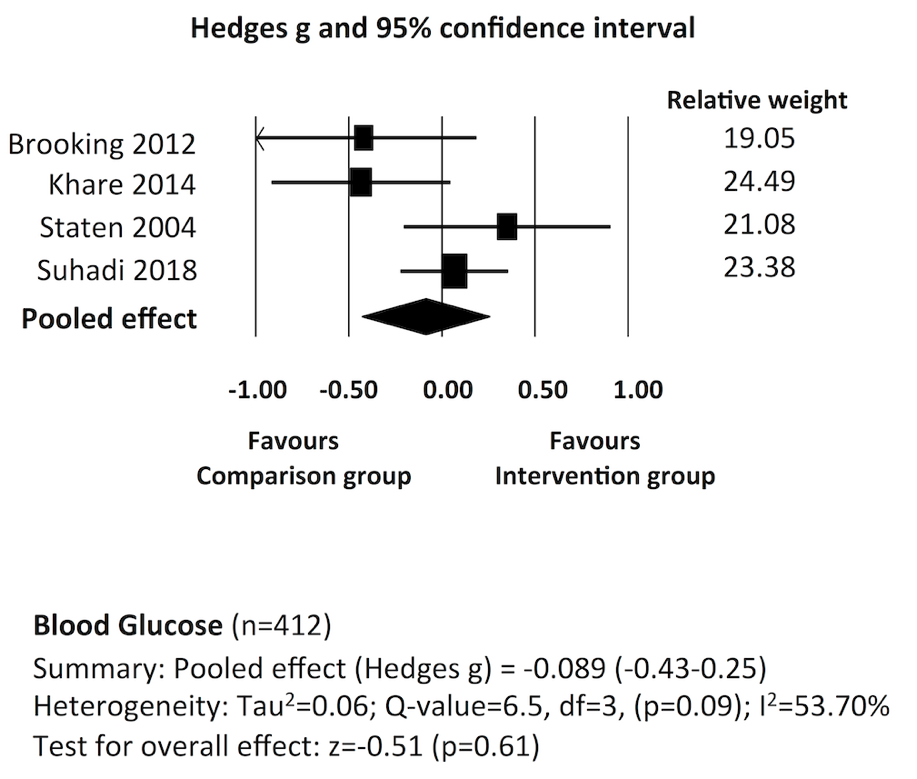


**Cholesterol**

Four of 11 studies were considered to have adequate methodological and clinical heterogeneity to pool results (n=439).(28, 30, 53, 57) We down-graded certainty in the evidence by four levels due to risk of bias, inconsistency (two levels), and imprecision. There is very low certainty that the pooled effect of educational interventions, when compared to minimal/no intervention, is 0.06 (95% CI = -0.43-0.55; Tau^2^ = 0.20) (figure d). The I^2^ value of 83% indicates a considerable degree of heterogeneity across study results.

**Figure d.** The effectiveness of educational interventions at improving total cholesterol in socio-economically disadvantaged populations: random effects meta-analysis


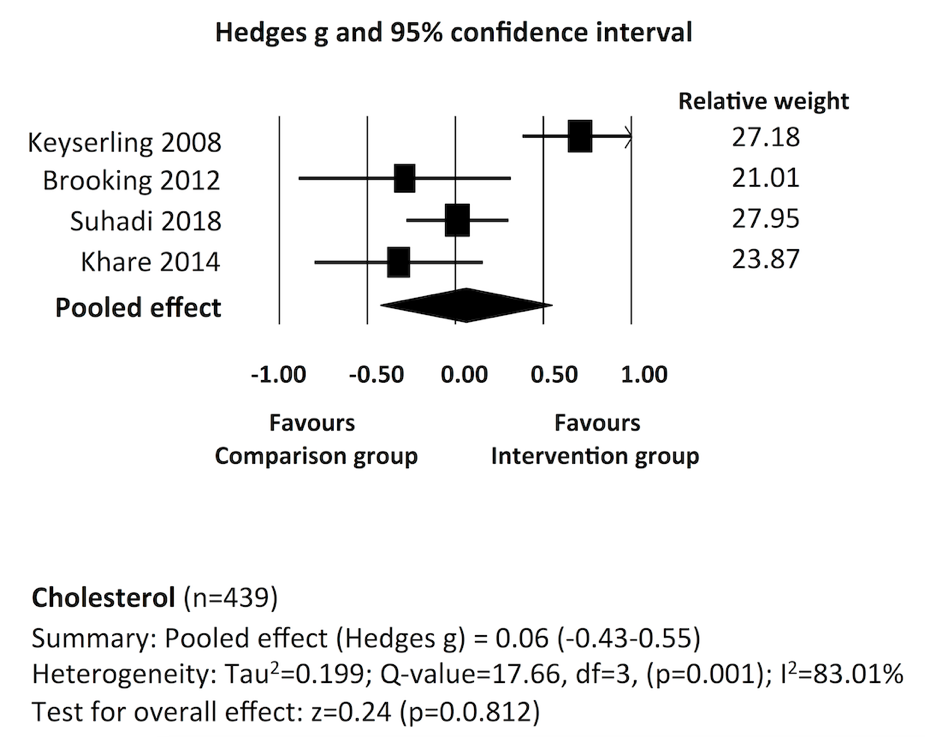

Supplement: Supplementary file 1 — Additional file 1. [file 12889_2023_15329_MOESM1_ESM.docx]
